# Trafficking of the NMDAR2B Receptor Subunit Distal Cytoplasmic Tail from Endoplasmic Reticulum to the Synapse

**DOI:** 10.1371/journal.pone.0039585

**Published:** 2012-06-27

**Authors:** Steve Standley, Ronald S. Petralia, Manneth Gravell, Rebecca Hamilton, Ya-Xian Wang, Manfred Schubert, Robert J. Wenthold

**Affiliations:** 1 Graduate College of Biomedical Sciences, Western University of Health Sciences, Pomona, California, United States of America; 2 Laboratory of Neurochemistry, National Institute on Deafness and Other Communication Disorders, National Institutes of Health, Bethesda, Maryland, United States of America; 3 Molecular Virology and Neurogenetics Section, National Institute on Neurological Diseases and Stroke, National Institutes of Health, Bethesda, Maryland, United States of America; Louisiana State University Health Sciences Center, United States of America

## Abstract

NMDA receptor NR2A/B subunits have PDZ-binding domains on their extreme C-termini that are known to interact with the PSD-95 family and other PDZ proteins. We explore the interactions between PSD-95 family proteins and the NR2A/B cytoplasmic tails, and the consequences of these interactions, from the endoplasmic reticulum (ER) through delivery to the synapse in primary rat hippocampal and cortical cultured neurons. We find that the NR2A/B cytoplasmic tails cluster very early in the secretory pathway and interact serially with SAP102 beginning at the intermediate compartment, and then PSD-95. We further establish that colocalization of the distal C-terminus of NR2B and PSD-95 begins at the *trans*-Golgi Network (TGN). Formation of NR2B/PSD-95/SAP102 complexes is dependent on the PDZ binding domain of NR2B subunits, but association with SAP102 and PSD-95 plays no distinguishable role in cluster pre-formation or initial targeting to the vicinity of the synapse. Instead the PDZ binding domain plays a role in restricting cell-surface clusters to postsynaptic targets.

## Introduction

*N*-methyl-*D*-aspartate (NMDA) receptor activation is necessary for associative learning and memory and induces long-term potentiation (LTP), a putative physiological substrate for memory [1], [2]. NMDA receptor activation has also been implicated in cell death associated with acute neurological disorders such as excitotoxicity and stroke-induced ischemia [3]. Changes in NMDA receptor function have been associated with chronic neurological and psychiatric disorders such as Huntington’s disease and schizophrenia [4], [5]. Thus, expression, trafficking, targeting and turnover of NMDA receptors have grown to be subjects of intense interest.

Functional NMDA receptors are heteromeric and typically contain two types of subunits, NR1 and NR2. NR1 subunits have eight separate splice variants, and most splice variants differ in the cytoplasmic C-terminal amino acid sequence [6], [7]. NR2 subunits are made up of four proteins encoded by 4 separate genes called NR2A-D [8]. NR2A and NR2B are highly expressed in the mammalian forebrain. Several lines of evidence support a central role for the C-termini of NR2 subunits in synaptic targeting of native NR1-NR2 heteromeric complexes. Truncation of nearly the entire C-terminus of NR2A in mice significantly diminishes appropriate synaptic targeting of NR1/NR2AΔC heteromers [9], indicating that the associated NR1 subunits combined with NR2A do not have sufficient sequence determinants for synaptic targeting. In primary cerebellar cortical granule cells, transfection of NR2B subunits lacking the distal C-terminal 7 amino acids (which contain the PDZ-binding domain) also results in the absence of incorporation of NMDA receptors into the synapse as measured by miniature NMDA receptor-mediated EPSCs [10], indicating that the NR1 subunits combined with truncated NR2B subunits in cerebellar cortical granule cells do not have sufficient sequence determinants for synaptic targeting. On the other hand, transfection of organotypic hippocampal slice neurons with NR2A or NR2B subunits lacking the last 6 amino acids significantly reduces, but does not abolish, synaptic targeting of NR1/NR2AΔ6 or NR1/NR2BΔ6 [11], indicating the combined NR1 subunit is incapable of rescuing the defect singularly.

NMDA receptor NR2 subunit cytoplasmic tails contain a PDZ-binding domain at the extreme C-terminus that can associate with all four members of the PSD-95 family of membrane-associated guanylate kinases (MAGUKs; PSD-93, PSD-95, SAP97, and SAP102), as well as with other MAGUKs and PDZ domain-containing proteins such as MALS [12], [13], S-SCAM [14], CIPP [15]. Imaging of NR2B transport vesicles in dendrites have revealed that NR2B-containing vesicles travel along microtubules and this transport appears to be at least in part mediated by the interaction with a multi-molecular protein transport complex comprised of the kinesin motor KIF-17, mLin-10, mLin-7, mLin-2/CASK and SAP97 [13], [16]–[18]. The PSD-95 family of proteins is notable for association specifically with the pre- or postsynaptic structures in neurons. Most evidence supports a role for the PSD-95 protein family association in maintaining or imparting localization of NR2 subunits to the synapse [for review, see [19]]. Thus, this interaction may underlie the essential function of the NR2 cytoplasmic tails in synaptic localization. However, evidence with two splice variants of the NR1 NMDA receptor subunit (NR1-3 and NR1-4; [20]–[22]), which contain a PDZ-binding domain and bind all PSD-95 family members, has suggested that associations between NR2 cytoplasmic tails and PSD-95 family may begin early in the secretory pathway and serve trafficking functions. SAP97 has also been shown to associate with the AMPA receptor subunit GluR1 early in the secretory pathway [23]. Notwithstanding these observations, there is still little known about the specific roles in trafficking that each individual PSD-95 family member might play. In particular, it remains to be determined which one(s) associate with which receptor subunits, or subunit combinations endogenously, under what conditions they associate, and where they associate with NMDA receptor subunits along the secretory pathway, and where and when they dissociate and under what conditions they dissociate. NR2A and NR2B share the same C-terminal 6 amino acids containing the PDZ-binding domain, but whether they associate with the same PDZ proteins, and whether those proteins play a role in the trafficking and targeting of NMDA receptors remains to be made clear. It is clear, however, that the final C-terminal 15 amino acids of both the NR2A or NR2B sequence can differentially influence synaptic localization [24], suggesting that more than just the PDZ-binding domain is at play in synaptic localization and protein-protein associations, at least at the synapse.

**Figure 1:**
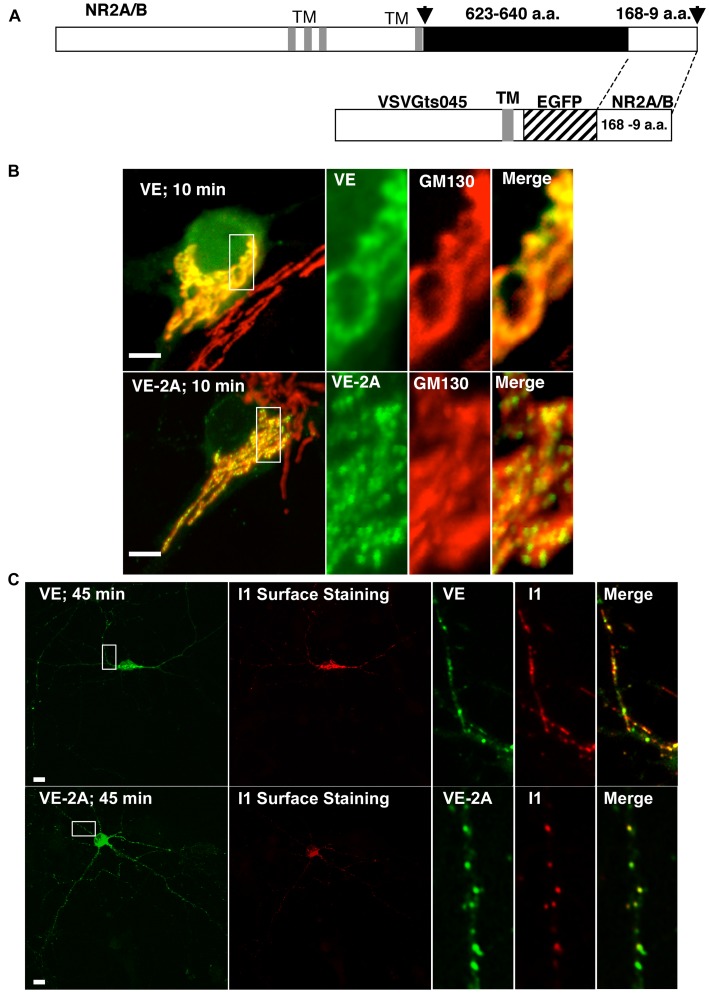
The NR2 cytoplasmic tails induced clustering early and throughout the secretory pathway. (A) The distal C-terminal one-third of NR2A and NR2B cytoplasmic tails was appended to the VSVGts045-EGFP in frame, beginning at the stop codon for EGFP (168 amino acids for NR2A and 169 for NR2B). (B) VE (top, left; scale bar 10 µm) 10 minutes after release from the ER co-localized with GM130, a *cis- medial*-Golgi marker. Panels to the right are enlargements of VE, and GM130, from left to right, respectively. Note the continuity of VE with GM130 at this stage in the secretory pathway and the lack of clustering. At the same time point, VE-NR2 chimeras (VE-2B not shown; VE-2A, bottom panels, left; scale bar 10 µm) mostly exhibited uniformly round clusters that also co-localized with GM130-defined Golgi cisternae. (C) GFP-fluorescence (top left panel; scale bar 10 µm) and i1 antibody surface staining (top panel, second from left) of VE 45 minutes after ER release (top panels) indicated surface targeting largely to the soma, and also at intervals to all neurites, extending the length of the processes without any preference for a particular one. Small, circular vesicles appeared in neurites; some exhibited surface staining while others were intracellular (see top right panels). However, 3 hours after release, VE surface staining was spread over the entire surface of the neuron (Fig. S2B, top right panel) and did not exhibit a clustered or organized geometric appearance in comparison to VE-2A/B. GFP-fluorescence (bottom left panel; scale bar 10 µm) and i1 antibody surface staining (bottom panel, second from left) of VE-2A (VE-2B shown in Fig. S2B at 3 hours permissive temperature) formed regular, organized clusters on the neuronal surface, similar to the *cis- medial-*Golgi co-localized VE-2A clustering at 10 minutes after ER release. Yellow lines extend along the regions enlarged in the right panels for VE (top) and VE-2A (bottom).

Physiological and immunocytochemical studies have provided the wealth of observations described above. However, standard transfection and immunocytochemical characterizations often are not able to examine dynamic aspects of cargo transport and targeting with definite knowledge as to where the cargo is located in or on the cell. For this reason, questions concerning the role of the NR2-PDZ protein interactions in the dynamics of receptor trafficking have been difficult to address. Therefore we created chimeric proteins consisting of a GFP-tagged temperature-sensitive Vesicular Stomatitis Viral Glycoprotein mutant, VSVGts045 [25], [26] and the distal cytoplasmic C-termini of NR2A or NR2B (Fig. 1). VSVGts045 is a transmembrane glycoprotein mutant that remains misfolded and retained in the ER at 40°C, but can rapidly fold and exit the ER upon shift to 32°C. Thus, by shifting temperature we are able to synchronize cargo exit from the ER and to observe the initial dynamics of endogenous protein-protein interaction during early cargo transport, when the NR2 and NR1-PDZ binding domain-containing cytoplasmic tails associate with their potential respective endogenous binding partners, and where the cytoplasmic tails are initially targeted.

Our observations of early trafficking events are made primarily by imaging the soma and proximal dendrites of neurons. Our observations of subsequent synaptic targeting events are made by imaging both distal and proximal dendrites. No clear pattern of differential SAP102 or PSD-95 antibody immunofluorescence could be observed in the soma and proximal dendrites without the VSVG-NR2A/B chimeras; only by synchronously releasing a wave of NMDA receptor C-termini were we able to discern clear differences along the secretory pathway among the potential interacting proteins. We find, to our surprise, that SAP102 appears to associate with NR2A/B subunits early after ER exit, significantly so at the level of the *cis-medial-*Golgi apparatus, but does not show the same colocalization with the PDZ-binding domain of the NR1 subunit, nor does the NR1-C2′ splice cassette-containing cytoplasmic PDZ binding domain induce clustering early in the secretory pathway. Our observations lead us to conclude that in spite of the general capacity of the PSD-95 family of MAGUK proteins for binding all of the PDZ binding domain-containing subunits of NMDA receptors in heterologous cells, these NMDA receptor subunit-MAGUK interactions are more specific, dynamic, and unpredictable than heterologous cell protein-protein interactions suggest. We further find, as was found previously much more profoundly in hippocampal neurons up until day 2 *in vitro* [27], that NR2B subunits further appear to colocalize with PSD-95 in clusters at the TGN. Our data suggest that co-transport of NR2B/SAP102/PSD-95 macromolecular clusters to the synapse may occur. However, our data are correlative in nature, and do not directly demonstrate this phenomenon. Our observations suggest that these well-established potential interactions are exquisitely controlled, and nuanced, when observed in neurons.

## Results

### ER Retention and Early Trafficking of VE and VE-NR2 Chimeras

The distal C-terminal segments [168 or 169 amino acids of rat NMDAR2A (2A) and NMDAR2B (2B), respectively] were subcloned in frame with VSVGts045-EGFP (VE) at the stop codon for EGFP (Fig. 1A). VE-2A and VE-2B chimeras retained the temperature-dependent trafficking characteristics of VE. We then confirmed the capability of PSD-95 to cluster VE-2B in COS-1 cells, as has been shown with full-length receptors [28]. This was confirmed also at the level of the ER when maintained at 40°C as well (Fig. S2A). Further, we verified that there was no leakage, or pre-existing cell surface population of VE using our transfection protocol and 24 hour, 40°C incubation (Fig. S3). Complete sensitivity to Endoglycosidase H (Endo H) when VE was maintained at 40°C for 24 hours indicated that all the VE cargo was distributed from the ER to, at most, *cis-*Golgi (see Fig. S3). In hippocampal neurons, VE-2A and VE-2B demonstrated diffuse intracellular fluorescence indistinguishable from VE (Fig. S1A) when maintained at 40°C for 18–24 hours.

Transfected hippocampal neurons (14 DIV) were incubated for 18–24 hours at 40°C, and the medium was exchanged with a medium equilibrated at 32°C. Time of medium exchange equals time zero, at which VE and VE-NR2 chimeras were allowed to exit the ER. VE, 10 minutes after ER exit, showed strong colocalization with the *cis-medial*- Golgi marker GM130 in the perinuclear region (Fig. 1B, top row). Note in particular the smooth continuity with the GM130 marker in the higher magnification micrographs. In contrast, VE-2A and VE-2B cytoplasmic tails demonstrated extensive clustering into small, regular, and circular patches that co-localized with perinuclear GM130 at the same time point (Fig. 1B, second row). After switching to 32°C, the VE constructs were immediately transported predominantly into the perinuclear Golgi apparatus. However, a minority of puncta appeared to remain in peripheral dendrites, as described previously using VSVGts045-YFP [data not shown; [29]]. We were not able to establish that the peripheral puncta co-localized with markers for the TGN, suggesting a small amount of transit through ‘satellite secretory pathways’ [30]. An important point to be considered is that we imaged the perinuclear Golgi region of the soma, and proximal dendrites, to observe early trafficking events. Since there is a large concentration of secretory cargo traversing this region, it afforded a robust signal, however it precluded observing more faint cargo, or distal dendrites. Thus, our imaging limitations could account for our inability to see secretory cargo in the peripheral dendrites traversing satellite secretory pathways. Indeed, recent evidence has indicated that secretory cargo emerging from the ER in neurons is spatially restricted by increased complexity at bifurcations and synapses in dendrites [31]. Thus, our observations should be tempered with the prospect that early secretion of spatially restricted cargo in distal dendrites might be different. Forty-five minutes after release from the ER, the leading edge of the pulse of cargo could be detected on the cell surface using the i1 antibody that recognizes an extracellular epitope of VE. We noted that VE was added to the surface abundantly on the soma and all neurites at regular intervals extending out from the soma to distal dendrites (Fig. 1C, top panels), eventually resulting in an even distribution over the entire neuronal plasma membrane (Fig. S2B, upper right panel). In contrast, VE-2A and VE-2B were added to the cell surface as circular clusters (Fig. 1C, bottom panels; also, see Fig. S2B, bottom right panel in comparison to the upper right panel). Clusters in one or more distal dendrites often appeared at more than 100 µm from the soma in the absence of comparable staining on the surface of the soma (data not shown), suggesting fusion of VE-2B cargo vesicles with the plasma membrane distal to the soma, although we cannot rule out vectored lateral diffusion from the soma. Recent evidence has also indicated that large AMPA receptor-containing recycling endosomes are exocytosed onto syntaxin 4-containing microdomains adjacent to postsynaptic specializations [32]. However, it remains to be determined whether emerging secretory NMDA receptor cargo is added in the same fashion. Surface expression of VE-2A and VE-2B clusters appeared unpredictable compared to VE, emerging variably on the soma or not, and on any one or more neurites. Thus, both NR2A and NR2B cytoplasmic tails induce the formation of clusters that undergo vesicle-mediated transport throughout the secretory pathway, and that appear to be added *en bloc* to the neuronal surface. Moreover, the limited and variable addition of VE-NR2 clusters to the plasma membrane compared to VE suggested that the distal C-termini of NR2 subunits of NMDA receptors imparted significant targeting and membrane fusion characteristics on the constitutively exocytosed VE reporter molecule.

### VE-2B Chimeras have Full-length NR2B Characteristics

**Figure 2:**
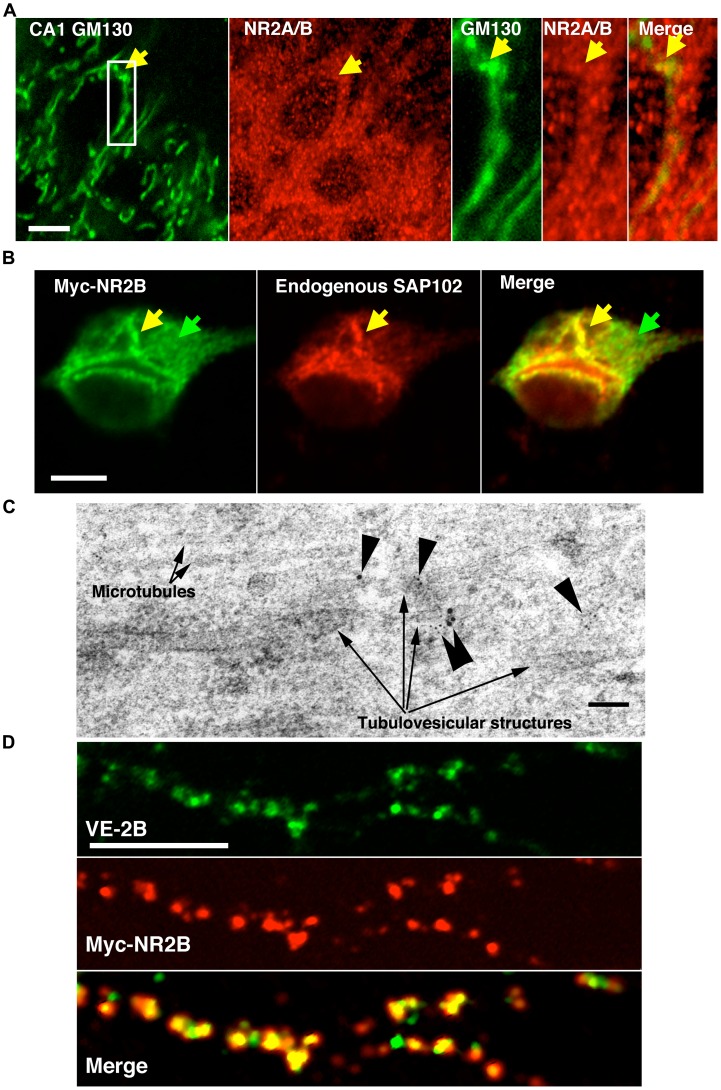
Relationship between native, full-length NR2s, and VE-NR2 chimeras. (A) Adult rat hippocampal CA1 pyramidal cells were immunostained with antibodies for GM130 (green) and NR2A/B C-termini (red). NR2 clusters co-localized with GM130 (yellow arrows), consistent with native receptor clustering early in the secretory pathway (scale bar 10 µm). (B) Full-length myc-tagged NR2B was transfected for 3.5 hours, and maintained at 20°C for 2.5 additional hours to block progress of myc-NR2B-NR1 beyond the TGN. Cycloheximide (100 µM) was added for the last 1.5 hours to reduce ER staining from recently synthesized myc-NR2B. The results shown above consist of a pulse of myc-NR2B-NR1 heteromeric receptors limited to between the ER and the TGN. Antibody staining for myc (left panel) and SAP102 (middle panel) demonstrated some clustering and co-localization of myc-NR2B with SAP102. Yellow arrows indicate co-localized puncta in the Golgi region, and green arrows indicate diffuse staining consistent with ER (scale bar 10 µm). (C) Immunogold labeling of intracellular NR2A/B (5 nm gold) and SAP102 (10 nm gold) along microtubules in the pyramidal cell body layer of hippocampal CA1 indicated co-localization of NR2A/B and SAP102, which was consistent with NR2A/B and SAP102 association early in the secretory pathway (scale bar is 100 nm). (D) VE-2B was transfected and the following day incubated for 24 hours at 40°C. Full-length myc-NR2B was serially transfected as described in (B) while neurons were incubated at 40°C. After 3 hours at 40°C, neurons were shifted to 20°C incubation for an additional 2.5 hours in the presence of Cycloheximide (100 µM) followed by 30 minutes at 32°C to allow both VE-2B and myc-NR2B to exit the TGN. The top panel shows VE-2B in a proximal dendrite targeted similarly to myc-NR2B (middle panel; scale bar 5 µm) in the same dendrite (bottom panel, merge).

To assess whether native NR2-NR1 heteromers appear as clusters early in the secretory pathway, 50 µm thin sections of adult rat brain were immunostained with antibodies to GM130 and NR2A/B. The staining pattern over the soma of adult (P60) rat hippocampal CA1 pyramidal cells appeared punctate, with puncta co-localized with GM130 (Fig. 2A). Hippocampal neuronal cultures were transfected with a myc-tagged full-length NR2B subunit and placed at 20°C to block progression through the TGN [33], [34]. Cultures were then immunostained with anti-myc and anti-SAP102 antibodies (Fig. 2B). The resulting distribution was limited to between the ER and TGN, and showed the beginnings of cluster formation in a perinuclear region consistent with the Golgi apparatus. Immunostaining also suggested that intracellular myc-NR2B was associated with SAP102 (Fig. 2B) as were VE-2A and VE-2B (see below). This was confirmed at the EM level by immunogold double labeling with anti-NR2B and anti-SAP102 antibodies (Fig. 2C). The picture in 2C was taken at the base of the apical dendrite of a CA1 pyramidal cell.

To assess whether VE-2B possessed a sufficient amount of the cytoplasmic tail to be targeted in a manner similar to that of full-length receptors, we used a serial transfection and temperature manipulation scheme. Myc-NR2B was transfected into hippocampal neurons previously transfected with VE-2B and maintained at 40°C. Three hours later, neurons were switched to 20°C for 2.5 hours and 100 µM cycloheximide was added to limit ER staining from newly synthesized myc-NR2B. Neurons were subsequently shifted from 20°C to 32°C media for 30 minutes to allow both VE-2B and myc-NR2B to exit the TGN. When exiting in this synchronized manner, myc-NR2B and VE-2B were colocalized in dendrites (Fig. 2D). This suggested that the distal C-terminal segment of NR2B contains some sequence determinants for appropriate targeting.

### NR2 Association with Different PSD-95-family Proteins Along the Secretory Pathway

**Figure 3:**
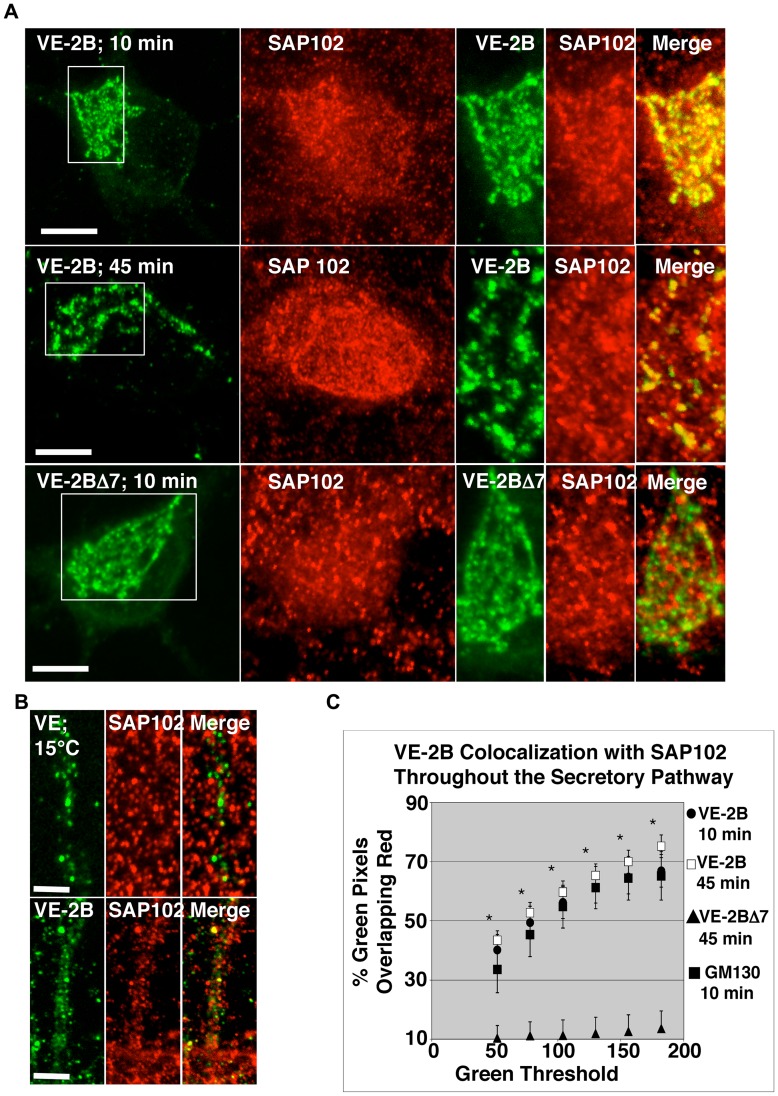
VE-2B clustered with SAP102 at 10 and 45 minutes after release from the ER. (A) At 10-minutes after ER exit VE-2B clearly co-localized with endogenous SAP102 (top panels, VE-2B EGFP fluorescence in green, and endogenous immunostaining with antibody to SAP102 in red; boxes depicts the enlargements; scale bars are 10 µm). At 45 minutes after ER exit (panels second from the top), a time point at which the leading edge of the pulse of VE-2B cargo is arriving at the cell surface (see Fig. 1C), endogenous SAP102 continued to exhibit strong colocalization. VE-2A demonstrated indistinguishable patterns of colocalization with SAP102 along the secretory pathway (summarized in Table S1). Colocalization of VE-2B with SAP102 is dependent on the distal C-terminal seven amino acid residues that contain the PDZ-binding domain of NR2B (bottom panels), as VE-2BΔ7 exhibited little colocalization with SAP102. Note that VE-2BΔ7 still exhibited apparent clustering (left panel at bottom; for quantification see Fig. 6E) and that endogenous SAP102 did not concentrate in the perinuclear Golgi region in the absence of bound receptor (bottom red panel). (B) Neurons transfected with VE, or VE-2B were incubated overnight at 40°C, then switched to media incubated at 15°C for 1 hour to block transport to the Golgi apparatus, and limit secretory cargo transport to no further than the IC. Neurons were then immunostained for endogenous SAP102. Concentration of VE was observed along the lengths of dendrites that was consistent with budding and protrusion from ER exits sites and initial transport to the Golgi (top panels; characterized previously [29]). However, concentrations of VE were not well colocalized with SAP102. Alternatively, concentrations of VE-2B demonstrated the beginnings of co-localization with SAP102 at the IC (bottom panels; scale bars 5 µm). (C) Colocalization was quantified as described in Experimental Methods with 5–15 images of neuronal soma from at least 3 separate transfection experiments for a minimum of 15 and a maximum of 26 measures. To assess whether concentrated clusters of VE-2B were co-localized with concentrated SAP102 clusters, the threshold of SAP102 was held constant at a threshold that included concentrated SAP102 clusters (104-255 gray level, inclusive), and the threshold of inclusion for VE-2B (green threshold) was varied from 52-255 to 182-255 (x-axis). The percent colocalization was averaged within transfection to reduce random error (the number of transfections equals N), and then within-group to obtain the means ± SEM. By one-way Anova with repeated measures, there was a significant group effect (P<0.01), and *post hoc* pairwise comparisons using the Tukey method that were significant are indicated by asterisks (p<0.05). E-2B was co-localized with SAP102 at both 10 minutes (filled circles), and 45 minutes (open squares) after ER exit. At 10 minutes after ER exit, VE-2B was also predominantly co-localized with GM130, a marker of *cis- media*- Golgi (filled squares). VE-2BΔ7 (filled triangles) showed incidental background levels of colocalization with SAP102. Similar results were obtained holding the green threshold at a high constant value and varying the red threshold.

Since the PSD-95 family of neuronal MAGUKs has been shown to cluster NR2 cytoplasmic tails, we reasoned that endogenous MAGUKs were likely to participate in the formation of the orderly clusters of VE-2A and VE-2B in neurons. As there were no significant differences between VE-2A and VE-2B by quantitative immunofluorescence with respect to any of the neuronal MAGUKs at any time point or condition analyzed after ER exit, colocalization data for VE-2A are only shown in Table S1. VE-2B transfected into hippocampal neurons were allowed to exit the ER for 10 minutes (Fig. 3A, top row) and 45 minutes (Fig. 3A second row), after which neurons were immunolabelled with an antibody specific to SAP102. Co-clustering of VE-2B with endogenous SAP102 was present at both time points. These results were confirmed with a second SAP102 specific antibody (Alomone Labs, data not shown). Quantitative analysis was performed by selecting high intensity SAP102 positive puncta (see Experimental Methods) and varying the threshold for VE-2B fluorescence from low to high. Results indicated that high intensity clusters of VE-2B were highly co-localized with SAP102 clusters (see Fig. 3C). Removal of the distal C-terminal 7 amino acids of VE-2B abolished its colocalization with endogenous SAP102, but had little effect on clustering (VE-2BΔ7; Fig. 3, bottom panels; quantified in Fig. 3C; clustering further quantified below). Early clustering of VE-2BΔ7 suggested that SAP102 may only be part of the early preassembly, and that other proteins could also participate in clustering, as proteins associated with domains other than the PDZ-binding domain of NR2s have been reported [for review; [19]]. SAP102 has also been shown to bind to the PDZ binding-domain of NMDAR1 splice variants, NR1-3 and 1–4 [20], when co-expressed in HEK293 cells. However, the NR1-3 cytoplasmic C-terminus appended to VSVG-EGFP demonstrated neither clustering nor co-localization with endogenous neuronal SAP102 clusters (Fig. S1B) at the level of the Golgi apparatus, suggesting that PDZ binding domain interactions may be far more selective in neurons.

Incubation at 15°C has been used to block secretory cargo transport to the Golgi apparatus and cause cargo to build up in the intermediate compartment [IC; for instance, see [35]]. We took advantage of this temperature blockade of transport to the Golgi apparatus to determine if SAP102 was capable of associating with NR2 as early as in the IC (Fig. 3B). VE transfected neurons subjected to 15°C medium exchange and incubation at 15°C for 1 hour demonstrated no colocalization with SAP102 clusters (Fig. 3B, top panels); however, VE demonstrated an appearance consistent with concentrating with COPII into budding vesicles and tubules [36]. In contrast to VE, VE-2B showed some specific colocalization with SAP102 under the same conditions (Fig. 3B, bottom panels). However, under 15°C incubation conditions for 1 hour, VE-2B cargo still appeared to be distributed between the ER and the IC, and the ER-localized VE-2B did not colocalize with SAP102.

**Figure 4:**
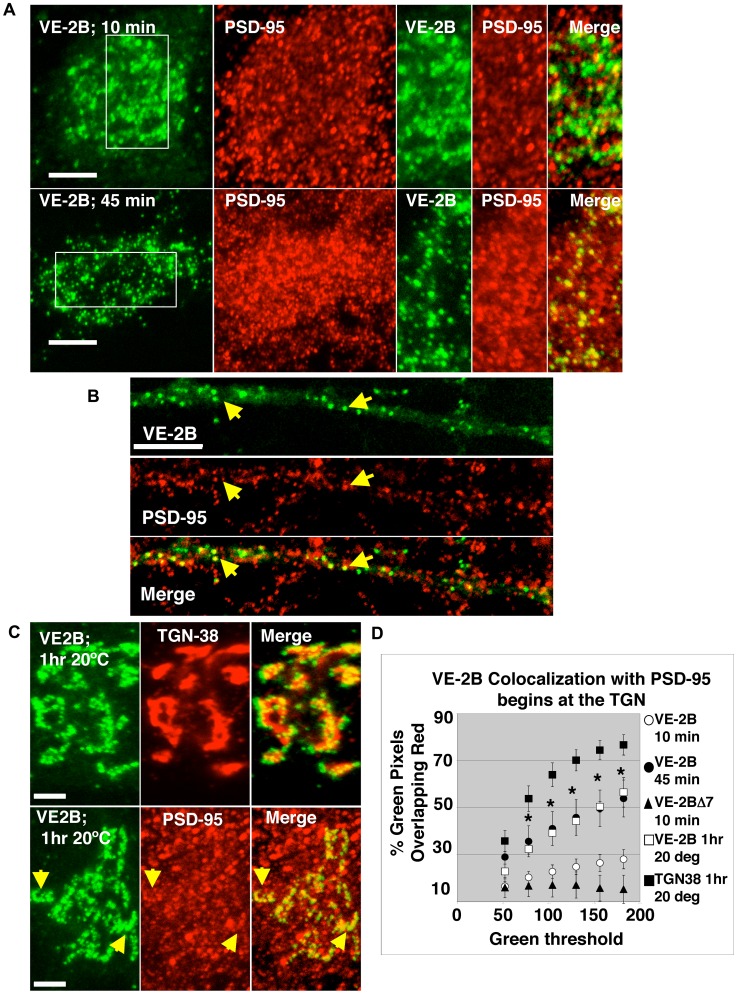
VE-2B co-localized with PSD-95 from the TGN to the plasma membrane. (A) At 10 minutes after release from the ER, VE-2B showed little colocalization with antibody staining to endogenous PSD-95 in the soma (top panels; scale bars are 10 µm). Forty-five minutes after ER release (second row), a time point at which the leading edge of VE-2B reaches the surface, PSD-95 showed significant colocalization with VE-2B. Similar results were obtained with VE-2A (see Table S1). (B) Colocalization with PSD-95 was also evident in dendrites at 45 minutes and later time points, such as 3 hours (see Fig. 6). VE-2B (top panel) showed co-localization with endogenous PSD-95 (middle panel) in dendrites at 45 minutes after permissive temperature, and later (scale bar 5 µm). (C) Neurons transfected with VE-2B were subjected to 40°C then the medium was exchanged with medium equilibrated at 20°C. Cultures were maintained at 20°C for 1 hour to allow VE-2B cargo exiting the ER to build up in the TGN. Immunostaining for endogenous TGN38 (a TGN marker; top panels) and PSD-95 (second row) in VE-2B transfected neurons subjected to the 20°C temperature manipulation showed robust colocalization (scale bars 5 µm). (D) Quantification of PSD-95 co-localization at different stages of the secretory pathway. There was a significant difference among the groups using a one-way Anova. However, *post hoc* comparisons demonstrated an insignificant pairwise difference at 10 minutes following ER exit. PSD-95 showed levels of co-localization with VE-2B (open circles) not significantly different from those of VE-2BΔ7 (filled triangles) 10 minutes after ER release. Forty-five minutes after ER exit, VE-2B co-localization with PSD-95 (filled circles) was significantly enhanced at most thresholds when compared to VE-2B colocalization at 10 minutes after ER exit. The significant increase in colocalization of VE-2B with PSD-95 seen at 45 minutes after ER exit could be reproduced by switching media to 20°C for 1 hour (open squares). To verify that VE-2B was concentrated in the TGN after 1 hour 20°C incubation, colocalization of VE-2B with TGN38 was quantified (filled squares).

Co-localization experiments with VE-2B and antibodies to PSD-95 indicated that PSD-95 did not associate with NR2 at early time points such as 10 minutes after release from the ER (Fig. 4A, upper panels), and quantification indicated that PSD-95 colocalization was not significantly different from background levels defined by colocalization with VE-2BΔ7 (Fig. 4D). At 45 minutes after ER release, VE-2B exhibited increased colocalization with PSD-95 (Fig. 4A, second row of panels; quantified in Fig. 4D). While from 0–10 minutes following ER release all constructs condensed into the perinuclear region, at 45 minutes VE-2B puncta were observed spread out along proximal and distal dendrites. This indicated a transition from processing through the central Golgi apparatus to transport in at least proximal dendrites. VE-2B colocalized with PSD-95 in dendrites as well (Fig. 4B). Quantification of colocalization of VE-2B with PSD-95 at 10 and 45 minutes was performed over the soma, and the results showed a significant increase in colocalization at 45 minutes, as compared to 10 minutes after release at most thresholds (Fig. 4D; p<0.05; see Experimental Methods for a description of the statistical methods used). To assess whether PSD-95 may associate with VE-2B before it reaches the cell surface, we took advantage of the well-characterized temperature blockade of exit from the TGN [33], [34]. When VE-2B was released from the ER at 20°C for 1 hour (Fig. 4C) and collected in the TGN (as assessed by the TGN marker TGN38), association with PSD-95 was significantly increased, as compared to its colocalization at 10 minutes (quantified in Fig. 4D, and pictured in 4C).

**Figure 5:**
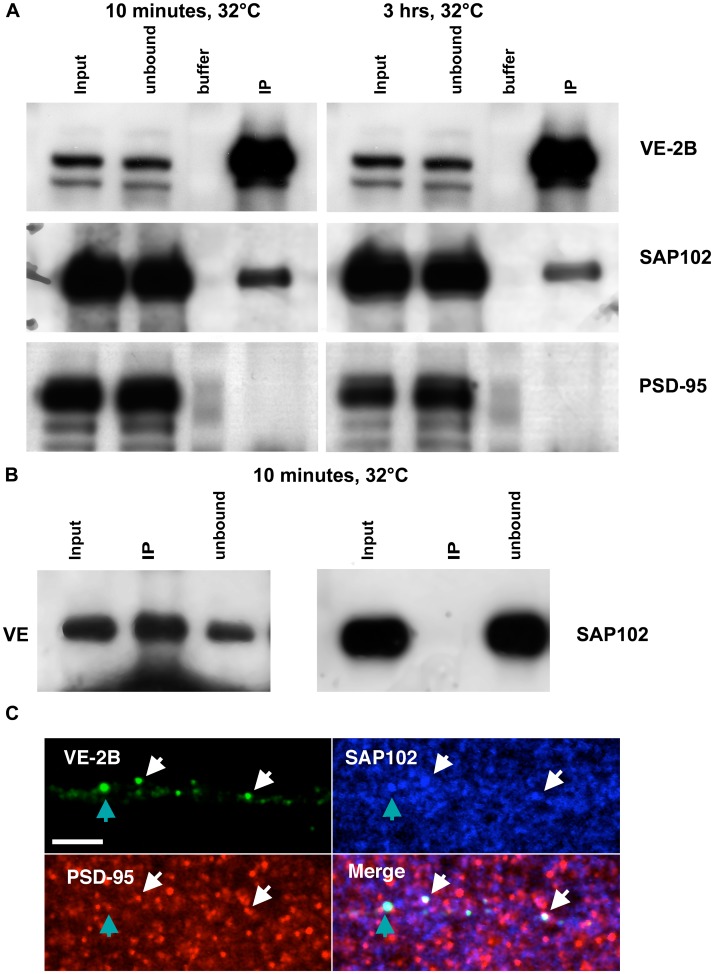
VE-2B interacted directly with SAP102, and VE-2B, SAP102 and PSD-95 formed co-clusters. (A) Western blotting was performed on cortical neurons infected with VE-2B and immunoprecipitated with i1 antibody to VSVG at 10 minutes and 3 hours after switch to permissive temperature (24 fold enrichment). Immunoblots were performed with rabbit anti-VSVG, PSD-95, and SAP102. Input, unbound, and immunoprecipitation fractions (IP) were run for each blot. To minimize the possibility of false-positives, buffer only was added to the lanes adjacent to the VE-2B IP lanes. Identical film exposure times for both anti-PSD-95 and anti-SAP102 indicate that SAP102 associates at 10 minutes and 3 hours after ER exit whereas PSD-95 does not. Longer film exposure times did not unambiguously indicate that PSD-95 was directly associated. (B) Western blotting was performed on cortical neurons infected with VE and immunoprecipitated with antibody to VSVG at 10 minutes after switch to permissive temperature. Immunoblots were then performed with rabbit anti-VSVG, and SAP102. Input, unbound, and IP fractions were run for each blot. The results indicated that VE (left panel) did not co-immunoprecipitate measurable quantities of SAP102 (right panel). (C) VE-2B transfected neurons 3 hours after ER release were immunostained for PSD-95 (red) and SAP102 (blue; scale bar, 5 µm). Aqua-colored arrows indicate VE-2B co-localized with SAP102 primarily. White arrows indicate VE-2B co-localized with both PSD-95 and SAP102.

To determine whether VE-2A or VE-2B directly interacted with SAP102 and/or PSD-95 at different points along the secretory pathway, we constructed Lentiviral vectors containing VE, VE-2A, and VE-2B to improve chimera expression in neurons. Hippocampal and cortical neurons transfected with VE, VE-2A, and VE-2B showed indistinguishable properties when examined by immunofluorescence to those described following calcium phosphate transfection (data not shown). Because of the larger amount of neurons obtained from cortical cultures as compared to hippocampal cultures, cultured cortical neurons (14 DIV) were transfected with a Lentivirus containing VE and VE-2B, held at 40°C for approximately 24 hours, and switched to 32°C to trigger release from ER for 10 minutes or 3 hours. VE and VE-2B were then immunoprecipitated using the i1 antibody. Input, unbound, and immunoprecipitate fractions were probed with antibodies to VSVG, SAP102 and PSD-95. SAP102 was preferentially coimmunoprecipitated with VE-2B over PSD-95 (Fig. 5A) at both 10 minutes and 3 hours after release, as was previously indicated by quantitative immunofluorescence in transfected neurons (compare Fig. 3C with Fig. 4D). Immunoprecipitation of VE at 10 minutes demonstrated no SAP102 co-immunoprecipitation (Fig. 5B). Neither VE-2B nor VE-2A immunoprecipitation resulted in substantial amounts of PSD-95 (VE-2A data not shown). However, immunofluorescence staining for VE-2B, SAP102, and PSD-95 simultaneously at 3 hours demonstrated the presence of both PSD-95 and SAP102 in the same puncta in many cases (Fig. 5C). Thus, while PSD-95 may become part of the VE-2A and VE-2B complexes as early as the TGN, these complexes may not interact directly with PSD-95 at the time points we assessed. A substantial amount of VE-2A, VE-2B and PSD-95 was insoluble in the 1% deoxycholate solution we used for immunoprecipitation, and therefore it remains quite possible that these proteins do associate directly and that the interacting proteins may be detergent insoluble.

### Synaptic Targeting of NR2A and NR2B Cytoplasmic Tails

**Figure 6:**
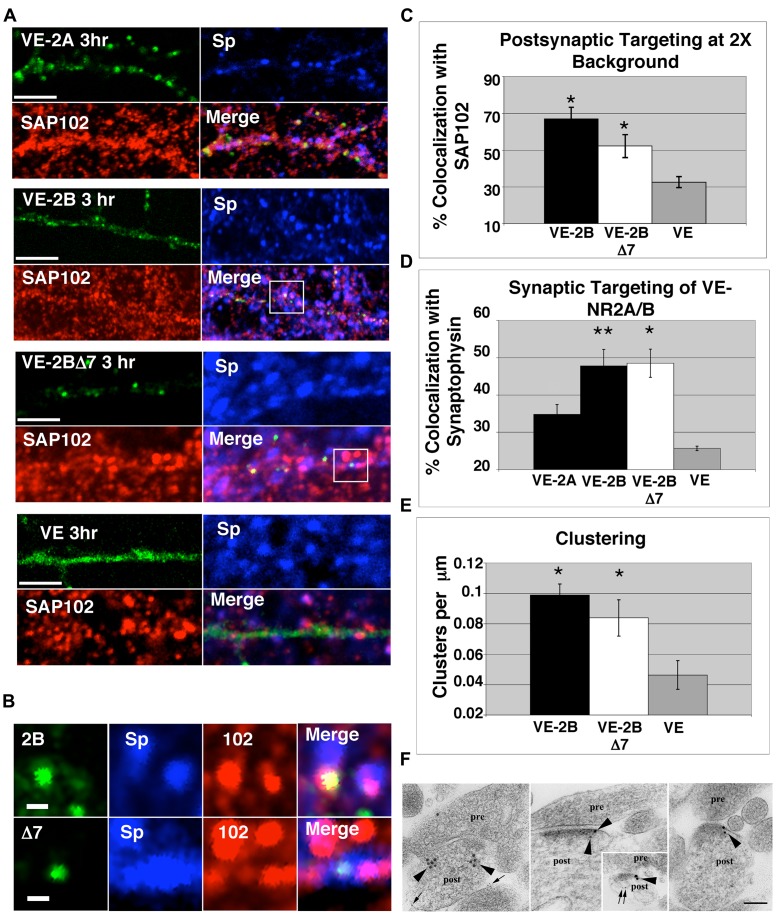
Targeting to pre- and postsynaptic markers. (A) Examples of VE-2A, VE-2B, VE-2BΔ7, and VE three hours after exit from the ER immunostained for SAP102 (red), and synaptophysin (Sp; pseudocolored blue), and merged. VE-2B, and VE-2BΔ7 demonstrate significant clustering compared to VE (quantified in E), and targeting to synaptophysin (quantified in D). (B) Higher magnifications of clusters from VE-2B and VE-2BΔ7 seen in A (indicated by boxes) demonstrate roughly equivalent colocalization to SAP102 (quantified in C) and synaptophysin (quantified in D; scale bars 1 µm). (C) VE-2B and VE-2BΔ7 co-localized with postsynaptic SAP102 at 2X background (52–255 inclusive gray scale). By one way Anova (P<0.01) there was a significant group difference. Using Tukey’s *post hoc* pairwise comparisons, both VE-2B and VE-2BΔ7 pixel overlap with SAP102 was significantly greater than VE (p<0.05), however VE-2B and VE-2BΔ7 were not significantly different than eachother. This relationship between VE-2B and VE-2BΔ7 overlap with SAP102 was only obtained when analyzing pixel overlap at 2X background. (D) The percent of overlap of VE-2B, and VE-2BΔ7 with synaptophysin was significantly greater than VE (one-way Anova with *post hoc* pairwise comparisons to VE * p<0.05). Surprisingly, VE-2A was not significantly different than VE among the 4 groups in a *post hoc* comparison. VE-2B targeted to synaptophysin significantly better than VE-2A (** p<0.05) but no differently than VE-2BΔ7. These relative differences were the same regardless of the green or blue threshold. The same results were obtained at this time point after ER release using synapsin as the presynaptic marker (data not shown). (E) Clustering was measured using Zeiss LSM510 image analysis software. Average intensity was calculated from each intensity graph of 20–30 dendrites for a total of 839.5 µm (VE-2B), 750.4 µm (VE-2BΔ7), and 776.0 µm (VE). A cluster was defined as being more than twice the average intensity of each dendrite for equal to or greater than 0.4 µm. The average number of clusters per µm ± SEM is plotted in E. There was a significant effect of group by one-way Anova. *Post hoc* comparisons indicated Both VE-2B and VE-2BΔ7 showed significantly more clustering than VE (p<0.05), and were not significantly different from each other. (F) Examples of immunogold labeling with i14 α-VSVG antibody (10 nm; arrowheads) and α-NR2A/B antibody (5 nm; arrows) indicate localization of VE-2B at synapses 3 hours after release from the ER (pre, presynaptic terminal; post, postsynaptic process). Scale bar is 100 nm. Quantification of 10 nm gold indicated that 10 of 47 synapses were labeled within 0–100 nm, and 18 of 47 (38.3%) synapses showed immunogold labeling within 0–500 nm of the postsynaptic density.

Analysis of surface targeting of VE-2A and VE-2B constructs indicated that, while the leading edge of surface delivery began at 45 minutes after ER exit, most clusters remained intracellular at this time (see below). At 3 hours following ER exit, more robust surface staining of VE-2A and VE-2B was evident (see Fig. S2B for example); therefore, we chose 3 hours after ER release to first examine synaptic targeting. VE-2A, VE-2B, and VE-2BΔ7 exhibited clustering and colocalization with SAP102 compared to VE (compare examples in Fig. 6A). Percent pixel overlap with SAP102 (Fig. 6C) or synaptophysin (Fig. 6D) was quantified with all thresholds at 2x background and the results indicated that VE-2B, and even VE-2BΔ7 staining overlapped with SAP102 and synaptophysin staining significantly more than VE (* one-way Anova with pairwise post hoc comparisons using the Tukey method; p<0.05). Percent overlap of VE, VE-2B, and VE-2BΔ7 staining with synaptophysin staining was calculated across a range of thresholds (green, 52–255 to 182–255; blue 52–255 to 110–255) and demonstrated the same relative relationship (data not shown). However, significant VE-2BΔ7 colocalization with SAP102 compared to VE was only observed at 2x background (see below). Clustering was assessed as described in Fig. 6E and demonstrated that both VE-2B and VE-2BΔ7 clustered significantly more than VE, and not significantly different than eachother. Examples of VE-2B and VE-2BΔ7 clusters co-localized with both SAP102 and synaptophysin in Fig. 6B illustrate the similarity of post- and presynaptic targeting. It also hints at one of the differences between VE-2B and VE-2BΔ7, namely that VE-2BΔ7 does not seem to co-transport with the MAGUKs to postsynaptic sites. Notice that the intensity of SAP102 co-localized with VE-2BΔ7 is lower than that of SAP102 associated with VE-2B (Fig. 6B). Cultures infected with Lentivirus containing VE-2B were processed for immunogold labeling 3 hours after ER exit. Immunogold labeling with antibodies to a VSVG epitope confirmed that a substantial number of the total synapses counted were labeled both directly, and also within 500 nm of a postsynaptic density (38.3%; see Fig. 6F).

Note that VE-2A showed less pixel overlap with synaptophysin than VE-2B (Fig. 6D), and was not significantly different than VE. This clear difference was unexpected, and seemed counterintuitive since NR2A subunits were thought to be enriched at the synapse [for review, see [19]]. However, evidence has indicated that synaptic targeting of NR2A-containing receptors is regulated, and not just a *de facto* consequence of NR2A expression [11]. In spite of VE-2A and VE-2B showing similar colocalization patterns with both SAP102 and PSD-95, and possessing the same extreme C-terminal 6 amino acids, their capacity to be targeted to synaptic markers was significantly different. This also suggested that specific association with SAP102 or PSD-95 via the PDZ binding domain was not playing a defining role in targeting VE-2A to the vicinity of the synapse.

**Figure 7:**
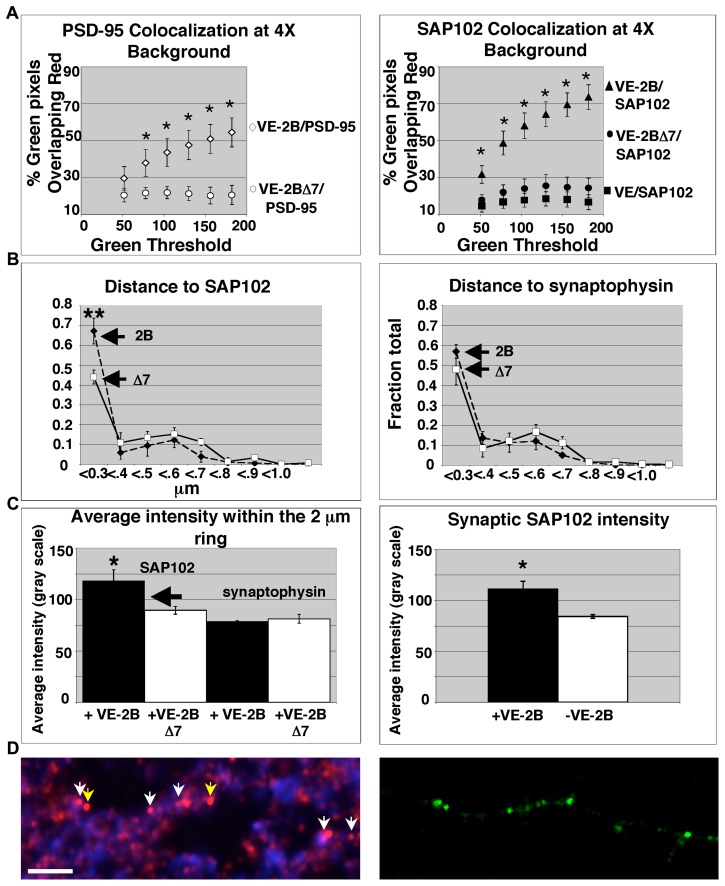
VE-2B and the MAGUKs are consistent with co-transportation to the synapse. (A) VE-2B chimeras co-localized with high intensity PSD-95 (left graph) and SAP102 (right graph) in dendrites 3 hours after release with the red threshold fixed at 4X background (104–255 inclusive gray scale). Colocalization was quantified as described in Experimental Methods. VE, VE-2B, and VE-2BΔ7 colocalization was assessed across the range of inclusive thresholds for green indicated on the x-axis. There was a significant group effect by one-way Anova even at even the lowest green threshold (p<0.05). VE-2B colocalization with SAP102 was significantly greater across all green thresholds using an Anova with repeated measures compared to both VE and VE-2BΔ7 (p<0.01), while the percent overlap of VE-2B with PSD-95 by Anova with repeated measures was significantly different than VE-2BΔ7 (p<0.01). (B) The distances between GFP, SAP102, and synaptophysin were obtained at 3 hours after release from the ER by defining a 2 µm region of interest around GFP puncta, and calculating the centroid for each color within the region of interest above 2X background using Metamorph software. Lengths are binned as <0.3 µm (unresolvable) to >1 µm in 100 nm increments as shown above. Results are shown as a fraction of the total puncta in all bins for 3 separate transfections with >50 GFP puncta per transfection ±SEM. A significantly greater percentage of VE-2B puncta are within an unresolvable distance to SAP102 compared to VE-2BΔ7 (B, left graph; p<0.05; one-way Anova with repeated measures), whereas there is no significant difference in the unresolvable groups of VE-2B, VE-2BΔ7, and synaptophysin (B, right graph). (C) Synaptic association of VE-2B is associated with an increase in SAP102 intensity. The mean intensity was calculated for all SAP102 and synaptophysin puncta within the 2 µm region of interest from the data pool in B. The resulting measure included both synaptic and non-synaptic VE-2B/SAP102. VE-2B-associated SAP102 staining showed a significantly higher mean intensity than VE-2BΔ7-associated puncta (C, left histogram; * p<0.05, Student’s t-test), while the mean synaptophysin intensity remained unchanged (C, left histogram). The mean intensities for the entire images from the data pool were the same. The arrow indicates the threshold at which SAP102 was included (104–255) for measurement of VE-2B/high-intensity MAGUK pixel overlap in A. To assess the effect of VE-2B on synaptic SAP102 (right histogram), the mean intensity of SAP102 puncta co-localized with both VE-2B and synaptophysin (<0.3 µm from each other) was extracted from a larger data pool (N = 4 transfections; total synaptic puncta, 289) and compared to the mean intensity of synaptic SAP102 in the same image pool and in the absence of VE-2B (total synaptic puncta, 699). Synaptic VE-2B significantly enhanced synaptic SAP102 intensity (*p<0.05, Student’s t-test; illustrated in D). (D) A typical example of how VE-2B (right picture, green) concentrated both synaptic and non-synaptic endogenous SAP102 (left picture, SAP102 in red; synaptophysin in blue) 3 hours after ER release (scale bar, 5 µm). Note that those SAP102 puncta that are co-localized with VE-2B (all arrows) are brighter than SAP102 puncta in the rest of the field. Yellow arrows indicate non-synaptic VE-2B/SAP102 puncta. White arrows indicate synaptic VE-2B/SAP102/synaptophysin puncta.

When we analyzed VE-2B, VE-2BΔ7, and VE for pixel overlap with high intensity PSD-95 and SAP102 (red, thresholded at 4X background; Fig. 7A), as was performed to assess co-localization along the secretory pathway (Figs. 3 and 4), VE-2BΔ7 no longer showed significant overlap with SAP102, as compared to VE (Fig. 7A, right graph). VE-2B showed significantly greater overlap with PSD-95 and SAP102 at 4x background than VE-2BΔ7 (* p<0.05; Anova with repeated measures; Fig. 7A, left and right graphs, respectively).

To determine VE-2B clusters that were transported along dendrites were associated with SAP102, we compared the distances between VE-2B, VE-2BΔ7, and both SAP102 and synaptophysin. We calculated a centroid for each color within a 2 µm region of interest (a ring) placed around GFP puncta for both VE-2B and VE-2BΔ7 as described in Fig. 7B. The distances from GFP centroids to SAP102 and to synaptophysin centroids were calculated. Our results indicated that VE-2B puncta were significantly more likely to be localized at the <0.3 unresolvable (colocalized) distance from SAP102 puncta (Fig. 7B, left graph; Student’s unpaired t-test of the <0.3 groups of 2B and 2BΔ7). VE-2B and VE-2BΔ7 showed no significant difference in the <0.3 groups with respect to synaptophysin (Fig. 7B, right graph). These results are consistent with our colocalization results at 10 minutes and 45 minutes after ER exit, and suggest that VE-2B is not only co-localized with SAP102 at synapses, but may be co-transported with SAP102. However, we do not show this directly.

Mean intensities of all puncta for SAP102 and synaptophysin within the 2 µm ring were quantified for VE-2B and VE-2BΔ7 (Fig. 7C, left histogram). There was a significant difference in mean intensity of SAP102 puncta in the VE-2B group compared to VE-2BΔ7 (Fig. 7C, left histogram, indicated) and no difference in mean intensity of synaptophysin (Fig. 7C, left histogram, indicated). Recall that colocalization with SAP102 high intensity puncta was quantified with the threshold for inclusion of SAP102 at 4X background (Fig. 7A). The arrow in Fig. 7C, left histogram, indicates that threshold. As was the case with all images used to quantify colocalization, there was no difference in the mean intensity of any color when we assessed the entire fields (VE-2B, 8.3±2.7 gray scale; synaptophysin, 43.6±6.9; SAP102, 37.8±7.9; VE-2BΔ7, 9.0±3.3; synaptophysin, 45.1±7.0; SAP102, 41.8±11.9). The mean intensity obtained in Fig. 7C, left histogram, includes both synaptic and non-synaptic VE-2B colocalized with SAP102. In order to determine if VE-2B and higher –intensity SAP102 were coincident at synaptic sites, we selected only SAP102 puncta localized <0.3 µm from VE-2B and synaptophysin and determined the mean intensity of SAP102 (Fig. 7C, right histogram). We found that synaptic VE-2B fluorescence was accompanied by an increase in mean intensity of synaptic SAP102 compared to synaptic SAP102 in the same fields that was not localized with VE-2B (* p<0.05). A typical example of VE-2B concentrating SAP102 with localization to synaptic sites is shown in Fig. 7D. Notice that VE-2B-colocalized SAP102 puncta are the brightest in the field regardless of non-synaptic (yellow arrows) or synaptic localization (white arrows).

### Surface Targeting of NR2 Chimeras

**Figure 8:**
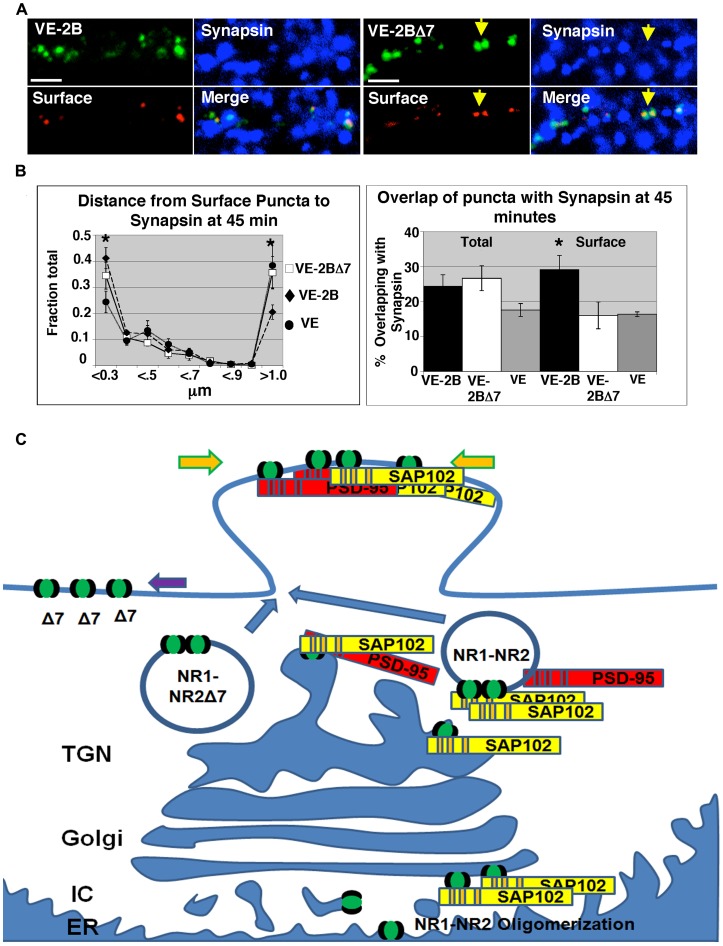
Surface targeting distinguished VE-2B from VE-2BΔ7. (A) VE-2B and VE-2BΔ7 were allowed to exit the ER for 45 minutes and then immunostained for surface expression with I1 antibody and presynaptic terminals with anti-synapsin. Yellow arrows in VE-2BΔ7 indicate surface puncta not in the vicinity of synapsin (right panels). At 45 minutes after ER exit, only about 30% of VE-2B and VE-2BΔ7 puncta in dendrites showed any immunostaining with i1 antibody. (B) The VE and VE-2BΔ7 surface puncta more than 1 µm away from synapsin were significantly greater in relative number than VE-2B (left panel). The surface VE-2BΔ7 within 0.3 µm is similar to VE-2B but not significantly different from VE, while VE-2B within 0.3 µm is significantly different than VE (one-way Anova considering VE, VE-2B, and VE-2BΔ7 in the >1.0 micron bins and then in the <0.3 micron bins, then pairwise *post hoc* comparisons; p<0.05). Centroids and distances were calculated with images thresholded at 2X mean background. Percent pixel overlap of green puncta with synapsin in the same data set showed no difference in the total VE-2B and VE-2BΔ7 at any threshold and trended toward increased synaptic localization at 45 minutes after permissive temperature, but did not reach significance when compared to VE, as was apparent at 3 hours (one-way Anova, p = 0.11; right panel, indicated as ‘total’). Green and red images from the same data set also were merged and color-thresholded for yellow to define the surface population. Percent overlap of yellow puncta with synapsin (blue) was then assessed for VE, VE-2B, and VE-2BΔ7 (right panel, indicated as ‘surface’). VE-2BΔ7 surface pixel overlap with synapsin trended toward a decrease compared to VE-2B at 2X background but not significantly until thresholded at 3X background (one-way Anova, *post hoc* comparison p<0.05). (C) Model of trafficking of NR2B. NR2B forms hetero-oligomers with NR1 subunits at the level of the ER [38], but the NR2 distal C-terminus is necessary and sufficient to confer significant synaptic localization [9], [11]. NR2A/B clusters with SAP102 early in the secretory pathway, and significantly so at the level of the *cis-medial-* Golgi. PSD-95 is added as part of the NR2B/NR1-SAP102 complex as soon as the TGN. NR2B/NR1-SAP102 complexes may be cotransported to the vicinity of the synapse, and also cotransported at least in-part along dendrites via Kif-17, mLin-2/Cask, mLin7, mLin10, and SAP97 in a poly-protein complex [see [18]] and added to postsynaptic structures. The NR2B/SAP102/PSD-95 association does not appear to be essential for immediate synaptic targeting, but is required for maintenance of position on the synaptic surface.

Because the PDZ binding domains of both NR2A and B are known to be important for synaptic localization as measured by electrophysiological responses in transfected neurons and since we could find no difference in the overall targeting of VE-2B and VE-2BΔ7, we explored how the PDZ binding domain might affect surface expression by examining antibody surface staining of VE-2B and VE-2BΔ7 at 45 minutes after ER release. Using a 2X background criterion, only about 30% of VE-2B (31.7±3.1%) and VE-2BΔ7 (34.2±6.4%) puncta were expressed on the cell surface. Total VE-2B, VE-2BΔ7, and VE percent pixel overlap with synapsin, another presynaptic marker, trended toward the same differences that were obtained at 3 hours after ER exit with synaptophysin (Fig. 8A, examples; quantified in Fig. 8B, right panel). Distances from surface puncta to synapsin were then assessed separately for VE-2B, VE-2BΔ7, and VE at 2X background as described for Fig. 7B (Fig. 8B, left panel) and the results indicated that surface VE-2B and VE-2BΔ7 puncta were similarly targeted to synapsin. However a significant portion of VE-2BΔ7 surface clusters were more than 1 µm away from synapsin, and the mean intensity of surface expression did not differ between VE-2B and VE-2BΔ7 (VE-2B, 16.3±0.9; VE-2BΔ7, 17.4±0.9 gray scale; p = 0.34). This suggests that while the intracellular and surface targeting is similar, VE-2BΔ7 surface clusters are unable to maintain location at or near the synapse. We sought to confirm this result with the same data pool (VE-2B, N = 7, 72 total images; VE-2BΔ7, N = 6, 53 total images; VE, N = 6, 51 total images) by examining pixel overlap. Surface (red) and total (green) puncta were thresholded at 2X and 3X background (2X, 52-255; 3X, 78-255 gray scale) and overlaid. Images were then color-thresholded to select for yellow and pixel overlap with synapsin at 2X and 3X was assessed. The results confirmed that VE-2BΔ7 surface puncta showed a marked decrease in colocalization with synapsin while the total pixel overlap was no different from that of VE-2B (Fig. 8B, right panel).

## Discussion

In this study we used chimeras of VSVG and C-terminal segments of NR2A and NR2B to explore trafficking of NMDA receptors in neurons. While significant amounts of information on NMDA receptor trafficking have been obtained using subunits tagged with GFP or other markers, a major limitation of this approach is the inability to identify the compartments with which the receptors are associated and the uncertainty of the origin of the receptors analyzed. Newly synthesized receptors cannot be distinguished from receptors destined for degradation. Since its exit from the ER can be controlled by temperature, VSVGts045 overcomes these limitations and offers the major advantage that the chimera can be analyzed at specific points along the secretory pathway. Using this approach our results showed an association of NMDA receptors with endogenous MAGUKs early in the secretory pathway, although SAP102 and PSD-95 associated at different locations. VE-2B and SAP102 colocalization appeared as early as the IC, and VE-2B colocalization with PSD-95 appeared as early as the TGN. Note, however, that we did not repeat all of the same experiments with VE-2A, so it is conceivable that VE-2A and VE-2B differ to some degree with respect to where they begin to associate with SAP102 and PSD-95. A chimera of VSVG containing the distal one-third of the C-terminus of NR2B was significantly targeted to synapses, supporting previous data that synaptic trafficking determinants are contained within the distal C-terminus of NR2B. VE-2A, on the other hand, while trending toward synaptic targeting, was not observed to be significantly greater than VE control. We found that NR2/MAGUK interactions were not necessary for the initial trafficking of the NR2B to the vicinity of the synapse, but that the MAGUK interaction appeared to be necessary to stabilize receptors on the cell surface near the synapse. These data are further supported by earlier findings in cerebellar granule cells that the PDZ-binding domain was necessary for restriction to the synapse, but not total surface expression [37], and by a recent study live-imaging indicating the PDZ-binding domain plays a direct role in maintaining NMDA receptors at or near synapses [24].

### NR2s are Necessary and Sufficient for Synaptic Targeting

When NR2 subunits are transcribed, translated, and translocated into the lumen and membrane of the ER, they oligomerize with NR1 subunits [38]. However, several studies have demonstrated that synaptic localization is dependent on the C-terminus of NR2A [9] and specifically the distal C-termini [11] of NR2A and NR2B, while a parallel role for NR1 subunits in synaptic targeting is not yet apparent, or appears thus far at least not to be able to rescue a synaptic targeting defect in combination with a truncated NR2A/B. We took advantage of these findings that determined the necessity of NR2 for synaptic targeting and focused specifically on the roles of NR2 distal C-termini in directing both specific protein-protein interactions and immediate targeting to synaptic sites. Our results clearly indicated the NR2B cytoplasmic C-terminus is not only necessary, but sufficient for immediate and significant synaptic targeting. Although the targeting of VE-2A under basal conditions is inconsistent with the greater synaptic targeting of NR2A/NR1 complexes [39], [40], it is consistent with the need for prior synaptic activity for synaptic targeting of NR2A. Indeed, while the cytoplasmic C-termini play a key role in synaptic localization, and are necessary and sufficient for synaptic targeting, additional structural elements appear to contribute to differential synaptic targeting of NR2A/NR1 or NR2B/NR1 heteromultimers. For instance, the glycosylation site in the B-loop of NR2B appears to differentially drive NR2B into the synapse under conditions of inactivity [41].

On the other hand, our conclusions are also made with some degree of caution, since there are four different splice-variants of the NR1 cytoplasmic C-terminus, two of which encode C-terminal PDZ binding domains as well as PDZ binding domain-embedded, di-valine, exit signal motifs [NR1-3 and NR1-4; [20], [42]]. In spite of earlier indirect evidence suggesting SAP102 may bind to and suppress the ER retention signal of NR1-3 beginning at the level of the ER [18], contrary evidence suggesting that the extreme C-terminal di-valines of NR1-3 and NR1-4, acting as exit signals, may abrogate ER retention have raised doubt about the former conclusion [42]. Thus, absent direct evidence, the precise role of NR1 C-terminal splice variants in trafficking and protein-protein interactions remains unknown and unpredictable.

### SAP102 Associates with the NR2 C-terminus Early in the Secretory Pathway

SAP102 and PSD-95 are normally found in abundance as part of the molecular scaffold at the postsynaptic density [43]. SAP102 can also be found abundantly in rat brain microsomal fractions [20], which are enriched in smooth ER membranes, Golgi and endosomal vesicles. Much less PSD-95 is found in this fraction. Immunogold localization of PSD-95 in rat brain hippocampal CA1 neurons does, however, indicate the presence of PSD-95 in association with intracellular organelles [27].

Our data indicated that at least some VE-NR2 chimeras were associated with endogenous SAP102 as early as the IC, however it is not clear if significance is reached until the *cis-medial-*Golgi. We observed this colocalization throughout the entire secretory pathway and at synaptic loci. In fact, VE-2B chimeras appeared responsible for delivering additional SAP102 to synapses, since the mean intensity of SAP102 staining increased specifically in the synaptic locations where VE-2B chimeras had been delivered. However, we do not show this directly by live-imaging, nor do we know if the additional SAP102 proteins become a permanent part of the postsynaptic architecture.

Several studies have indicated that PDZ proteins interact at the level of the ER [[20]–[22], [44], [45]], and play an important role in the early trafficking of membrane proteins. PDZ protein interactions appear to be required for ER exit in the case of pro-TGFa [44] and of the NMDA receptor subunits, NR1-3 [20]–[22]. Also SAP97 has been shown to interact with the AMPA receptor, GluR1 subunits, in greater abundance before the *medial*-Golgi than on the cell surface [23]. Full-length NR2B subunits remain in the ER in the absence of oligomerization with NR1 subunits [45]. In COS-1 cells, NR2B transfected without NR1, which remained in the ER, was shown to associate with endogenous SAP102 and co-transfected SEC8 at the level of the ER [45]. However, no such interaction at the level of the ER has been demonstrated between NR2B and endogenous SAP102 in neurons. Moreover, PDZ binding-containing NR1 splice variants (NR1-3 and NR1-4) have been shown to directly interact by coimmunoprecipitation experiments with all of the PSD-95 family of MAGUKs, including SAP102, in HEK293 cells [20]. Prior work also indicated that SAP102 may be the MAGUK associated with endogenous NR1 subunits in rat brain microsomal fractions, which contain ER membranes and Golgi vesicles [20]. However, no observable co-localization between VE-NR1-3 and SAP102 was found early in the secretory pathway in neurons (Fig. S1B). In COS-1 cells, VE-2B did show colocalization at the level of the ER with co-transfected SAP102, or co-transfected PSD-95 (Fig. S2A). However, the earliest we were able to observe any qualitatively reasonable co-localization between VE-2B and endogenous SAP102 in neurons was at the level of the IC. Also, we were able to observe significant colocalization and direct interaction by coimmunoprecipitation between VE-2B and SAP102 as early as the *cis- medial-* Golgi apparatus in neurons. Together, these studies point to specific pre-assembly of NMDA receptor NR2A/B subunits and SAP102 complexes early in the secretory pathway. However, it is unclear precisely where these interactions begin in neurons, and what other proteins are integrated into these pre-assembled complexes.

### PSD-95 Associates with NR2B-SAP102 Complexes at the TGN

VE-NR2 association with PSD-95 was not significantly higher than controls when VE-NR2 chimeras were co-localized with the *cis*-, *-medial-*Golgi marker GM130. If VE-2B cargo was allowed to exit the ER and concentrate in the TGN, PSD-95 showed significant colocalization. Therefore, in all likelihood, either recycling or newly synthesized PSD-95 begins to associate with emerging NR2B cargo in significant quantity at the TGN; our data did not discriminate between the former two PSD-95 pathways. However, cotransfection of VE-2B with PSD-95-dsRED2 in neurons showed earlier association of VE-2B and PSD-95 (unpublished observations). This suggested that the VE-2B/PSD-95 colocalization we observed may be recycled PSD-95. However, we did not directly demonstrate such recycling. We were not able to coimmunoprecipitate substantial amounts of PSD-95 with VE-NR2s at any point up to three hours after exit from the ER. This result could be interpreted in several ways. First, PSD-95 could be added to VE-NR2/SAP102 containing clusters but not interact directly with VE-NR2s. Immunostaining for both PSD-95 and SAP102 with VE-2B indicated that some clusters contained both SAP102 and PSD-95, which supports the possibility that PSD-95 was not interacting directly with VE-NR2s. Alternatively, a substantial amount of VE-NR2 chimeras was insoluble in deoxycholate, leaving open the possibility that VE-NR2s interact directly with PSD-95 but that the complexes are insoluble. Yet a third alternative is that the NR2B-PSD-95 interaction requires a sequence in the C-terminus of NR2B that is not included in our VE-2B chimeras [46], [47]. This sequence, amino acids 1149-1157 of NR2B, is followed C-terminally by a long unfolded (i.e., intrinsically disordered) sequence that causes ER retention [[48]; and see [49]]. Including such a sequence on our reporter molecule may have resulted simply in ER retention; we chose to use a sequence that we were sure would traverse the secretory pathway. Thus, with this additional PSD-95-binding sequence, the direct interaction of NR2B with PSD-95 may be accomplished even without dissociation of the NR2B PDZ-binding domain from SAP102, and accomplished via a more proximal sequence on the NR2B molecule. In any case, colocalization with PSD-95 with our chimeras was dependent on the PDZ binding domain, as VE-2BΔ7 was not co-localized with PSD-95 at higher intensity thresholds. Thus, if the interaction is indirectly through the PDZ-binding domain it must still be accomplished through direct association with SAP102 or another interaction involving the PDZ binding domain of NR2B. A final possibility is that only a small fraction of VE-NR2B interacts with PSD-95 directly. Thus it would be too little to detect by co-immunoprecipitation experiments.

Several functional aspects of the TGN highlight the importance of an interaction beginning there. The TGN has been shown to serve as a sorting station for newly synthesized cargo destined for different subcellular locations [for review, see [50]]. It is possible that the addition of PSD-95 to specific VE-NR2/SAP102 clusters imparts unique targeting or elaborates significantly on the molecular make-up of emerging clusters. Also, the TGN receives molecules that recycle through the endosomal pathway. It is possible that some subpopulation of PSD-95, removed from postsynaptic compartments, recycles to the TGN and associates with new cargos. Molecules recycled to the TGN such as TGN38 and furin contain tyrosine motifs essential for that recycling [see for instance [50]–[52]]. PSD-95 contains a functional endocytic tyrosine motif at the extreme C-terminus [53] that serves both synaptic targeting and endocytic functions that result in perinuclear localization. Moreover, transfected PSD-95 in heterologous cells shows substantial overlap with subcellular markers for both endosomes and TGN [27]. Thus, given the right subunit combination or ensemble of proteins, the tyrosine motif of PSD-95 could also be essential for recycling to the TGN in neurons, however PSD-95-mediated endocytosis and TGN targeting of NMDA receptors has been tried to some unknown degree, and has failed so far to be demonstrated [54], and it remains to be determined how our observations, and prior observations of NR2, NR1, and PSD-95 colocalization with TGN markers can be explained [27]. It is noteworthy, however, that prior studies of NMDA receptor trafficking which concluded that NMDA receptors undergoing transport were not associated with PSD-95 did not evidence the full scope of receptor subunit combinations [55], [56]; only dsRED or GFP tagged and transfected NR1-1a subunit movement has been observed relative GFP-tagged PSD-95, and there are 3 other splice variations of the NR1 C-terminus. Thus, there is ample untested scope to potentially observe abundant NMDA receptor/PSD-95 cotransport in neurons.

Another body of observations relating to synaptic versus non-synaptic NMDA and PSD-95 localization concerns the effect of activity on NMDA receptor localization. In particular, it was concluded that the effect of application of the NMDA receptor competitive antagonist, AP5, over several days to several weeks appeared to cause non-synaptic NMDA receptors to migrate to PSD-95-containing postsynaptic sites [57]. However, the same group later noted that not all NMDA receptors that colocalized with PSD-95 were synaptic [58], opening up the possibility of NMDA receptor/PSD-95 cotranport to the synapse. Indeed, under the same conditions of inactivity with either TTX or AP5, wherein an increase in *synaptic* PSD-95 colocalized NMDA receptors was observed [57], a significant *decrease* in both the number of synaptic puncta containing PSD-95, and a *decrease* in total PSD-95 that was unrelated to turnover was observed [59]. Also, application of AP5 to neuronal culture has been shown to radically alter the NR1 splice variant(s) expressed, and that the splice variant expressed with inactivity (NR1-C2’), is responsible for synaptic accumulation of NMDA receptors [42]. Considering the totality of the findings above with respect to AP5 and NMDA receptor trafficking, we believe there is considerable indirect evidence and scope to propose that synaptic PSD-95 may not simply remain at the synapse in perpetuity until it is discarded or until NMDA receptors migrate to it, but instead it could be removed and recycled back to the TGN to associate with emergent NMDA receptor cargo complexes better disposed to precisely associate with established presynaptic input. This would explain the effect of AP5 on both the increase in synaptic NMDA receptor localization and the decrease in synaptic PSD-95 by proposing a trafficking function for PSD-95 that is inherently related to activity. Thus, it is unlikely that prior observations of NR1-1 transport alone fully evidence the scope of NMDA/PSD-95 cotransporation, and therefore they are not necessarily in contrast to this study.

### Subunit-specific Differences in Synaptic Targeting

When we compared percent overlap of VE-2B, and VE, the VE-2B subunit cytoplasmic tail showed a significantly greater portion of their area overlapping with synaptophysin and synapsin than VE. VE was widely distributed and showed a predominantly smooth appearance with the exception of transport vesicles. While this represented random or indiscriminate targeting, VE did show substantial overlap with synaptic markers because it was so widespread. In contrast, both VE-NR2s formed clusters that were generally transported and restricted to areas co-localized with presynaptic and postsynaptic markers.

Although VE-2A percent pixel overlap with synaptic markers trended toward an increase compared to VE, it did not reach significance and was also significantly less than that of VE-2B (and VE-2BΔ7; Fig. 6D). This was unexpected. However in light of the fact that there were no measured differences between VE-2A and VE-2B in association with SAP102 and PSD-95, it also suggested that sequence determinants other than the PDZ binding domain influenced VE-2A targeting. Since the developmental transition from NR2B to NR2A-containing NMDA receptors is dependent on agonist binding to pre-existing surface NMDA receptors [11], proper targeting of newly expressed NR2A-NR1 is not simply a consequence of synthesis but an active regulation of the targeting process. Considering the requirement for pre-activation of NMDA receptors, VE-2A synaptic targeting may be dependent on prior NMDA receptor activity.

### The Role of the PDZ Binding Domain in NR2 Trafficking

It is well established that PSD-95 clusters NR2 subunits co-transfected into heterologous cell lines and that the clustering is dependent on the distal C-terminal PDZ binding domain [for review, see [60]]. In cultured hippocampal neurons from mutant mice with nearly the entire C-terminus of NR2A truncated, a significant reduction in clustering was observed [9]. By the criteria described in Fig. 6E, we found that VE-2BΔ7 chimera clusters significantly compared to VE. Moreover, the frequency of clustering was similar to that of VE-2B. This strongly suggests that other sequence determinants, and likely other protein-protein interactions, contribute to clustering NR2 molecules *in vivo*.

Several lines of evidence indicate that the PDZ binding domain of NR2 subunits is essential for precise synaptic localization [for review, see [19]]. Evidence from several groups indicates that truncation of the C-terminus (which includes the PDZ binding domain) reduces NMDA receptor-mediated evoked synaptic responses [9], [11], and has an even more profound effect on miniature excitatory postsynaptic currents [9], [10]. However, as previously noted [9], these experiments were unable to distinguish between impairment in transport, targeting, or anchoring of NMDA receptors.

Using VSVG chimeras we were able to determine more precisely among the three possibilities for impairment. The amount of VE-2B and VE-2BΔ7 surface staining was not different at 45 minutes after release from the ER, indicating that the lack of PDZ-binding domain does not have a significant effect on transport to, or exocytosis at the neuronal surface. Our results are consistent with VE-NR2 cargo being tightly regulated, but that the PDZ binding-domain does not play a dominant role in transport to synapses, or initial cell-surface targeting. Our data further support the role of the PDZ-binding domain as that of restricting NMDA receptors to the synapse [24], [37], and further suggest that it does not serve a primary role in determining where NMDA receptors are inserted into the plasma membrane as well.

Truncation of the PDZ binding domain of VE-2B (VE-2BΔ7) had no effect on the initial targeting to the vicinity of two different presynaptic markers (synapsin and synaptophysin). However, VE-2B synaptic targeting was significantly coincident with additional SAP102 and PSD-95 appearing in the postsynaptic area. Forty-five minutes after ER release, a minority of both VE-2B and VE-2BΔ7 puncta were on the cell surface (about 30% of GFP puncta). While the targeting of total VE-2BΔ7 was identical to that of VE-2B, the two chimeras could be distinguished by examining their smaller cell-surface pools. The surface pool of VE-2BΔ7 demonstrated both a significantly diminished percentage of pixel overlap with synapsin, and a larger population of surface clusters >1.0 µm away from synapsin. Note also from the distance graph in Fig. 8B that the relative quantity of surface VE-2BΔ7 puncta localized at <0.3 µm was not significantly different from that of VE-2B. Taken together these data indicate VE-2BΔ7 chimeras are initially targeted (even to the cell surface) appropriately, but lack the capacity to remain anchored in the vicinity of the postsynaptic structure (see summary model, Fig. 8C). We therefore confirmed that the MAGUK/NR2 protein association serves to anchor NMDA receptor complexes in the postsynaptic area.

### The NR2/MAGUK Interactions and the Construction of the PSD

Modularity of vesicles or packets destined to pre- or postsynaptic structures has been the topic of many live imaging studies [55], [61]–[65]. It has been shown that as many as ten different proteins that function at presynaptic active zones are preassembled into dense-core vesicles for transport to presynaptic terminals [62]. These preassembled modules apparently constitute a relatively fixed portion of the total proteins at a mature presynaptic bouton. In comparison, other work has indicated that both NMDA receptors and PSD-95 form mobile transport ‘packets’ [55] or ‘modules’ [63], [64]. Movement of PSD-95 modules has produced speculation that postsynaptic densities are preassembled [63]. However, the size and molecular composition of postsynaptic packets or modules remains unexplored.

Some evidence suggests that NR2B-NR1 heteromeric receptors may be linked to and transported by KiF17, a mLin-10 binding kinesin, via mLin-10/mLin-2/mLin-7 interactions [13], [16]. Additionally, KiF1ba has been shown to interact directly with PSD-95 and SAP97 [66]. While our data indicated that the bulk of transport appears to be in association with SAP102, it is possible that NR2B binds to mLin-7 and forms a subpopulation transported by KiF17. Another possibility is that KiF17 binds SAP102 as well as mLin10.

We identified at least two neuronal MAGUKs, SAP102 and PSD-95, that can be preassembled with NMDA receptors before addition to postsynaptic structures. This represents only a small fraction of proteins that have been identified at the postsynaptic density [67]. Based on these observations we can only assert that subcomponents of the postsynaptic structure are preassembled. Another possibility is that the MAGUKs that are associated early are serving a function limited to the transfer of NR2s to PSDs, and that the interaction is short-lived; this may even involve SAP102 and PSD-95 being recycled to an intracellular compartment for interaction with new emerging cargo. Yet another view is that NR2s might be responsible for directed transport of SAP102/PSD-95 to synapses. However, recent experiments examining recovery from photobleaching have demonstrated recovery of SAP102-GFP fluorescence within minutes [68]. This suggests that perhaps SAP102 is playing a broader role in delivering cargo to the postsynaptic area than one limited to delivery of NMDA receptor cargo. On the other hand, SAP102-associated NMDA receptor recycling cargo may be relatively free of compartmental specificity. It will be of great interest to determine which additional molecules NR2s might be directing through the secretory pathway and into the postsynaptic area. The collective observations that both pre- and postsynaptic components may preassemble suggest a general theme in neuronal protein trafficking: Preassembly could serve to rapidly secure fully functional nascent synapses, as well as provide a means for rapid, all-or-none-changes in the molecular (sub)-composition of developing synapses, such as the switch from NR2B-containing synapses to NR2A-containing synapses that occurs in early development in as little time as 2 hours [69].

## Materials and Methods

### Ethics Statement

All animal procedures used in this study were conducted according to the guidelines of the National Institutes of Health Animal Care and Use Committee.

### Antibodies

I1 hybridoma monoclonal antibodies to VSVG were a generous gift from Doug Lyles (Wake Forest), and have been characterized previously [70]. Rabbit VSVG antibody was a generous gift from Carolyn Machamer (Johns Hopkins), and has been characterized previously [71]. Two separate anti-SAP102, and anti-PSD-95 antibodies have been characterized previously (rabbit anti-SAP102, JH62514; rabbit anti-SAP102, Alomone Labs; rabbit anti-PSD-95, T60; mouse PSD-95, Transduction Labs; [43]. Anti-SAP97 monoclonal antibody was from Stressgen (Victoria, B.C.). GM130, and TGN-38, and PDS-95 monoclonal antibodies were acquired from BD Biosciences, Transduction labs (San Diego, CA). Rabbit anti-NR2A/B (T12) has been characterized previously [72]. Monoclonal anti-synaptophysin antibody was from Chemicon International, Inc. (Temecula, CA), and anti-rabbit synapsin from Synaptic Systems (Gottingen, Germany). Rabbit anti-calnexin antibody was from Abcam. Highly cross-adsorbed Alexa-555 and 633-conjugated mouse and rabbit anti-IgG antibodies from Invitrogen were used for both representative micrographs and quantitative measurements. These antibodies showed minimal cross-reactivity and no measurable bleed across channels.

### Constructs

The VSVGts045-EGFP (a generous gift from Nelson B. Cole, NHLBI, NIH) was subcloned into the PCDL vector via XhoI/EcoRV sites. An XbaI site was added in-frame, beginning at the stop codon for EGFP, by site-directed mutagenesis using a Quickchange kit from Stratagene. The last 168, and 169 amino acids of NR2A and NR2B, respectively and NR2BΔ7 cytoplasmic tails were appended to VSVG-EGFP (XbaI), via PCR with XbaI/EcoRV restriction sites. Correct cytoplasmic tails were confirmed by DNA sequencing. Because the NR1-3 cytoplasmic tail DNA sequence contains an EcoRV site, NR1-3 cytoplasmic tail (amino acids 859 to 944) was subcloned into only the XbaI site of VSVG-EGFP, and then sequenced for orientation and correct amino acid sequence.

### Cultures, Transfections, and Immunostaining

Hippocampal and cortical neuronal cultures were prepared and cultured in serum-free Neurobasal medium (Invitrogen, Carlsbad, CA) as previously described [53], except that no antibiotics were used. COS-1 (ATCC #CRL-1650) and HEK293T (a gift from Fred Gage, Salk Institute, La Jolla, CA) cells were grown in DMEM with 10% fetal calf serum and Gentamicin at 5 µg/ml. Transfection of VE constructs was performed using the calcium phosphate method as previously described [20]. Neurons were then washed extensively with DMEM (Invitrogen; Carlsbad, CA), placed back in conditioned Neurobasal and allowed to incubate overnight at 37°C. The following day, hippocampal and cortical neurons were placed in a 40°C incubation chamber (5% CO2) overnight. On the third day, Neurobasal media equilibrated to the appropriate temperature was substituted. Incubations were terminated at various time points after media substitution by fixation in −20°C methanol (MAGUK staining), or 4% paraformaldehyde in PBS (i1 surface staining). After two washes in 1X PBS, neurons were then blocked with 10% normal goat serum (Vector Laboratories) for 1 hour. Neurons were incubated with primary antibodies for 1 hour using the following dilutions: anti-GM130, 1∶200; anti-TGN38, 1∶200; anti-PSD-95, 1∶500 (T60); anti-PSD-95, 1∶150 (TL); anti-SAP102, 1∶500 (JH62514); anti-SAP102, 1∶200 (Alomone); anti-Synapsin, 1∶500; anti-Synaptophysin, 1∶500; anti-VSVG, 1∶3 (i1 hybridoma). After 3 successive washes in PBS, neurons were incubated with Alexa-555 (1∶1000), or Alexa-633 (1∶500) for 30 minutes. Neurons were then washed in PBS 3 times and mounted on slides using a Prolong̈ Antifade Kit (Molecular Probes).

### Lentiviral Infection

VE, VE-2A, and VE-2B transgenes were inserted into a third generation, self-inactivating lentiviral vector [73]. The particular genomic construct we used has been reported previously [74]. The genes were expressed under control of the cytomegalovirus (CMV) immediate early gene promoter. For the generation of vector particles, vector DNA was transfected into HEK293T cells together with several support plasmids encoding the structural and regulatory proteins of the vector. Vector particles were collected from the medium for three consecutive days, purified and concentrated by ultra-centrifugation. Subsequent infections of hippocampal neurons for immunogold labeling, or cortical neurons for immunoprecipitation, were carried out in small volumes of medium. Stable transduction of the cells and transgene expression was evaluated 24 hours later. Infected neurons were then subjected to temperature manipulations identical to those of neurons transfected for immunofluorescence microscopy. Tissue was harvested and prepared for either immunoprecipitation or electron microscopy.

### Confocal Microscopy

Transfected neurons were imaged using a Zeiss LSM510 scanning confocal microscope. Representative pictures were taken with a 63X (1.4 N.A.) PlanApo oil objective at 3X zoom and 1024x1024 pixels (each image 50 µm×50 µm), as stacks of 0.8 µm optical sections for each color track, scanned at 0.4 µm intervals, and presented as cropped projections of approximately 2–4 µm total. For quantitative microscopy, all pictures were taken with the same laser power settings across compared groups. The lower magnification images in Figs. 1C, S1A, and S2B were taken with a 40X (1.3 N.A.) Plan Apo oil objective at 0.7X zoom (each image 325 µm×325 µm), as projections of 2.75–4 µm, scanned at 0.55 µm intervals.

### Quantitative Analysis

Data collection for quantitative analysis was performed blind, except for colocalization with subcellular markers, such as GM130 and TGN38. Images taken for quantification of dual-color colocalization were all one optical section of 0.8 µm for each color track, the same objective and settings as above were used with the amplitude offset and amplitude gain held constant across compared groups. The signal gain was varied to obtain matched-intensity distributions across pictures. This allowed a standardized comparison of colocalization within each picture measurement. Each experimental condition measured contains 3 to 5 separate transfections and 5–25 repeated entire field measures per transfection. Means and standard errors were calculated by averaging within and then across transfections within experimental groups. For three-color measurements with VE, VE-2A, VE-2B, and VE-2BΔ7 (EGFP) with PSD-95 or SAP102 (Alexa-555) and synaptophysin (Alexa-633) single optical sections of 1.2 µm per color track were taken as indicated above for two-color measurements. The data pool consisted of three-color images of both proximal and distal dendrites captured in the absence of the neuronal cell body. Unless otherwise indicated, between 4 and 9 separate transfections were performed, with 5-20 repeated measures (pictures) for each transfection were averaged within transfection. Means and standard errors were calculated as above. For analysis of surface expression all settings were fixed across groups for the channel used to measure surface expression (Alexa-555) so that relative intensities could be compared.

Image analysis was performed using Metamorph (Universal Imaging). LSM files were converted to 2- and 3-color TIFF files. No significant differences in average intensity were observed across any groups. Percent of EGFP area overlapping with other colors was calculated (except Figs. 7 and 8; see figure legend). Colocalization was determined for comparison by using the same thresholds for each image in every compared group. Significance (P<0.05) was assessed using a two-tailed, unpaired Students’ t-test when two groups were compared. When more than 2 groups were compared, or when repeated-measures were taken into account, a one-way Anova was used, and subsequent pairwise *post hoc* comparisons were made using Tukey’s test with a 95% confidence interval. Statistical tests were performed using either Excel (Student’s T-test) or Minitab16.

### Electron Microscopy

Rat brains and hippocampal cultures (14 DIV) were treated with 4% paraformaldehyde/0.5% glutaraldehyde, and processed for immunocytochemistry as described previously [e.g., [72]]. Tissue was cryoprotected and frozen in liquid propane in a Leica CPC, then freeze-substituted into Lowicryl HM-20 in a Leica AFS. Thin sections were labeled with primary antibody, followed by 5 and/or 10 nm gold secondary antibody, and were stained with uranyl acetate and lead citrate

### Immunoprecipitation and Western Blotting

Immunoprecipitation experiments were performed as described previously [43]. Cortical cultures (14 DIV) were homogenized in 50 mM Tris-HCl, pH 8, containing Complete©, a protease inhibitor cocktail (Roche), and 1 mM EDTA. Membranes were solubilized in 1% deoxycholate (DOC), 50 mM Tris-HCl, 1 mM EDTA, pH 8, for 45 min at 37°C. Insoluble material was removed by centrifugation for 1 hour at 100,000xg. Triton X-100 was added to a final concentration of 0.1%. For immunoprecipitation, supernatant from i1 hybridoma culture was used. Protein G beads were then pelleted, washed in PBS plus 0.1% Triton X-100 and incubated with 1 ml of the DOC-solubilized tissue at 4°C with constant rotation. The beads were then washed with 50 mM Tris-HCl, pH 7.5, containing 0.1% Triton X-100 three times, resuspended in 2x SDS sample buffer and boiled for 3 min. Proteins were separated with SDS-PAGE and transferred to Immobilon-P membrane and treated as described [35]. Bands were visualized on Hyperfilm with ECL+plus (Amersham Biosciences). Endoglycosidase H experiments were performed as described previously [20].

## Supporting Information

Figure S1 **VE-NR2s were indistinguishable from VE at the level of the ER, and VE-2A/B clustering and SAP102 association after ER exit is PDZ binding-domain specific.** (A) VE (far right panel) and VE-2A (left and middle panels) both showed indistinguishable diffuse fluorescence in neurons that co-localized with ER markers (not shown), as has been described for VE previously [see [29], Fig. 1A] when maintained at 40°C. The left panel shows an entire VE-2A transfected neuron (scale bar 30 µm). The white arrow indicates the region enlarged for comparison in the center panel to a neurite of the same length from a VE transfected neuron maintained at 40°C. (B) VE-NR2 association and clustering with SAP102 is PDZ binding-domain specific. The entire NMDAR1-3 cytoplasmic C-terminus was appended to VE (VE-NR1-3; see Experimental Methods) and transfected into neurons. Transfected VE-NR1-3 neurons were maintained at 40°C, then switched to 32°C media for 10 minutes, and immunostained for endogenous SAP102 and GM130. The cytoplasmic tail of NR1-3, although having a similar PDZ binding-domain and the demonstrated capacity to bind SAP102 and all other members of the PSD-95 family of MAGUKs in co-transfected HEK293 cells (see [20], Fig. 7A), showed no co-localization with endogenous SAP102 in neurons (compare panels; the panel to the far right is an enlargement of the Golgi region of the Merge panel; scale bar is 20 µm) 10 minutes after release from the ER. All neurons that were examined exhibited the same lack of co-localization of VE-NR1-3 with SAP102 10 minutes after permissive temperature.
(TIF)

Figure S2 **Characterization of VE-NR2 chimeras.** (A) VE-2B and PSD-95 co-clustered in the ER in heterologous cells. VE-2B (upper left green panel; scale bar 25 µm) and PSD-95 (upper panel pseudocolored blue, fourth from the left most upper panel) were transfected into COS-1 cells and maintained at 40°C overnight, then immunostained with mouse anti-PSD-95, and rabbit anti-calnexin (CNX; upper red panel, third from the left). The large merged panel (far right panel, predominantly white, indicating 3-color colocalization) clearly indicated VE-2B co-clustered with both PSD-95 and Calnexin at the level of the ER. Moreover, the clustering appeared similar to prior examples of PSD-95 clustering at the plasma membrane [75]. VE-2B also co-localized with SAP102 when maintained at 40°C (lower four panels from left to right are VE-2B, followed by enlarged VE-2B, SAP102, and Merge). We noted, however, that SAP-102 did not induce clustering *per se*, but showed the same pattern of co-localization accumulated on the nuclear membrane and in intracellular perinuclear structures as has been previously noted when SAP102 was co-expressed with another receptor binding partner, Kv1.4, which resulted in an intracellular accumulation of both proteins, and an absence of surface targeting [75]. (B) VE is normally added in a constitutive fashion to the cell surface in other cell types (see for example, [76]), and appeared to exhibit the same characteristic in neurons. At 3 hours of permissive temperature, VE alone covered the entire surface of the neuron and exhibited a relatively equal distribution throughout (compare the left upper panel EGFP fluorescence of VE to the I1 surface staining in the upper right panel; scale bars, 10 µm). In comparison, much of VE-2B appeared to remain intracellular (compare the lower left panel to lower right panel), and the neuronal-surface VE-2B was limited to discrete clusters even after 3 hours at permissive temperature. VE and VE-2B images were processed in the same manner.
(TIF)

Figure S3 **VE is tightly restricted to the ER when maintained at 40**°**C for 24 hrs.** VE was transfected into COS-1 cells for 8 hours, then incubated overnight at 37°C (approximately 16 hours), then placed in a culture incubator equilibrated at 40°C for 24 hrs. Media was preincubated to 32°C, and then the 40°C media was rapidly switched out for the 32°C media. Trafficking of VE was terminated by placing the tissue culture plates on ice after 6 hours at 32°C. Plates were scraped of cells and prepared for Endoglycosidase H (Endo H) treatment and gel electrophoresis. Samples were loaded into a polyacrylamide gel with molecular weight standards, and transferred to membranes using standard methods. Note that the 40°C, 24 hour sample is completely Endo H sensitive (second band from the right). Note also that the small lower molecular weight band in the 32°C lane is a non-specific band that is seen at 32°C but not in samples incubated with or without Endo H at 40°C. Further, N-Glycosidase F treatment, which cleaves all forms of N-glycosyl moieties, migrates no lower than the Endo H-sensitive band (data not shown).
(TIF)

Table S1 **Summary of co-localization and targeting of VE constructs.** Percent overlaps with PSD-95, SAP102, GM130 and TGN38 are given at 4X background, while overlap of VE constructs with synaptophysin is given at 2X background. Asterisks indicate significance after one-way Anova, with post hoc comparisons using the Tukey method.
(DOCX)
