# Supplementary figures and images for: Trafficking of the NMDAR2B Receptor Subunit Distal Cytoplasmic Tail from Endoplasmic Reticulum to the Synapse
Source: PLoS One. 2012 Jun 27;7(6):e39585. doi: 10.1371/journal.pone.0039585 (PMC3384676; doi:10.1371/journal.pone.0039585)

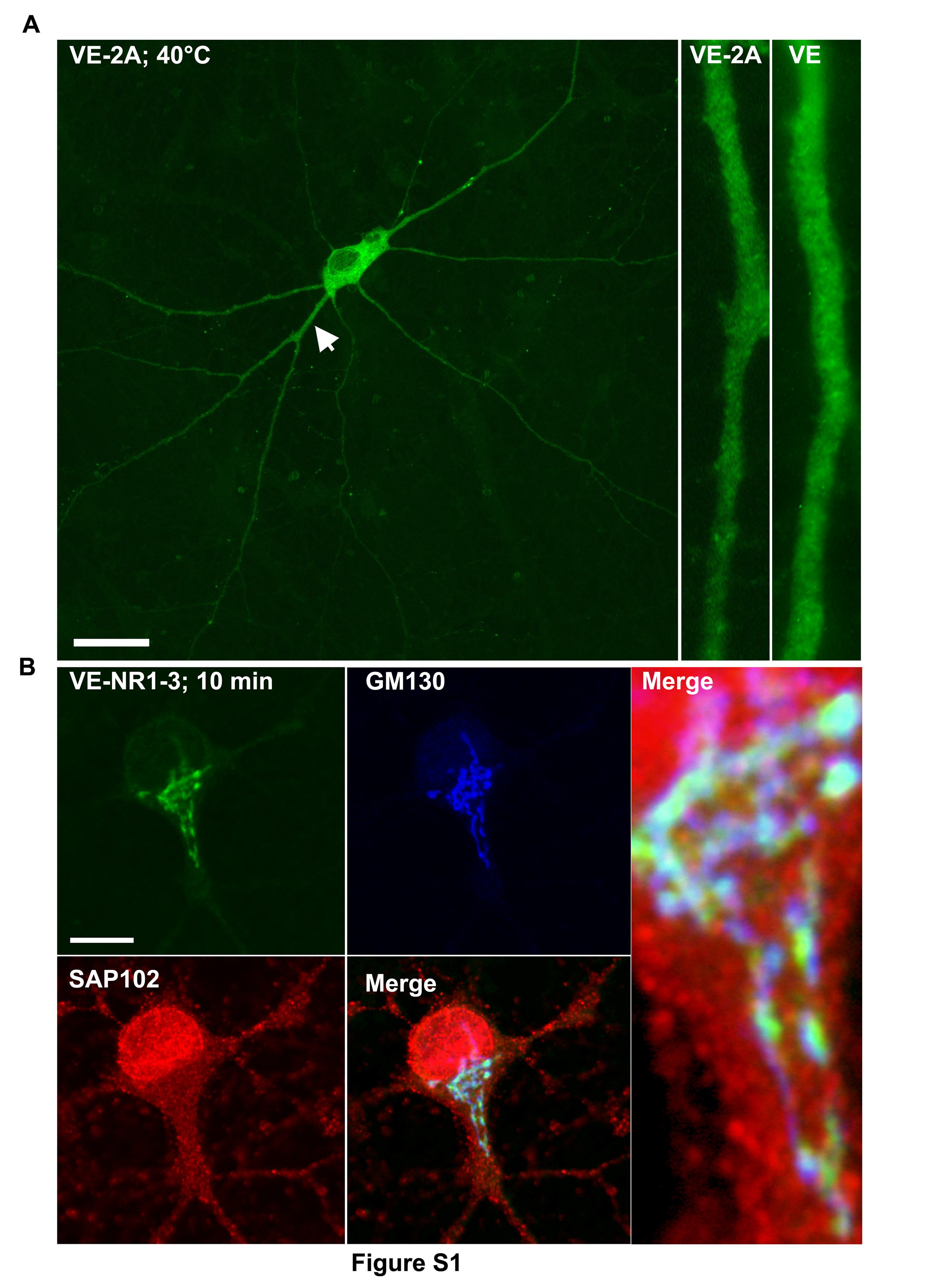

Supplement: Figure S1 — VE-NR2s were indistinguishable from VE at the level of the ER, and VE-2A/B clustering and SAP102 association after ER exit is PDZ binding-domain specific. (A) VE (far right panel) and VE-2A (left and middle panels) both showed indistinguishable diffuse fluorescence in neurons that co-localized with ER markers (not shown), as has been described for VE previously [see [29], Fig. 1A] when maintained at 40°C. The left panel shows an entire VE-2A transfected neuron (scale bar 30 µm). The white arrow indicates the region enlarged for comparison in the center panel to a neurite of the same length from a VE transfected neuron maintained at 40°C. (B) VE-NR2 association and clustering with SAP102 is PDZ binding-domain specific. The entire NMDAR1-3 cytoplasmic C-terminus was appended to VE (VE-NR1-3; see Experimental Methods) and transfected into neurons. Transfected VE-NR1-3 neurons were maintained at 40°C, then switched to 32°C media for 10 minutes, and immunostained for endogenous SAP102 and GM130. The cytoplasmic tail of NR1-3, although having a similar PDZ binding-domain and the demonstrated capacity to bind SAP102 and all other members of the PSD-95 family of MAGUKs in co-transfected HEK293 cells (see [20], Fig. 7A), showed no co-localization with endogenous SAP102 in neurons (compare panels; the panel to the far right is an enlargement of the Golgi region of the Merge panel; scale bar is 20 µm) 10 minutes after release from the ER. All neurons that were examined exhibited the same lack of co-localization of VE-NR1-3 with SAP102 10 minutes after permissive temperature. (TIF) [file pone.0039585.s001.tif]

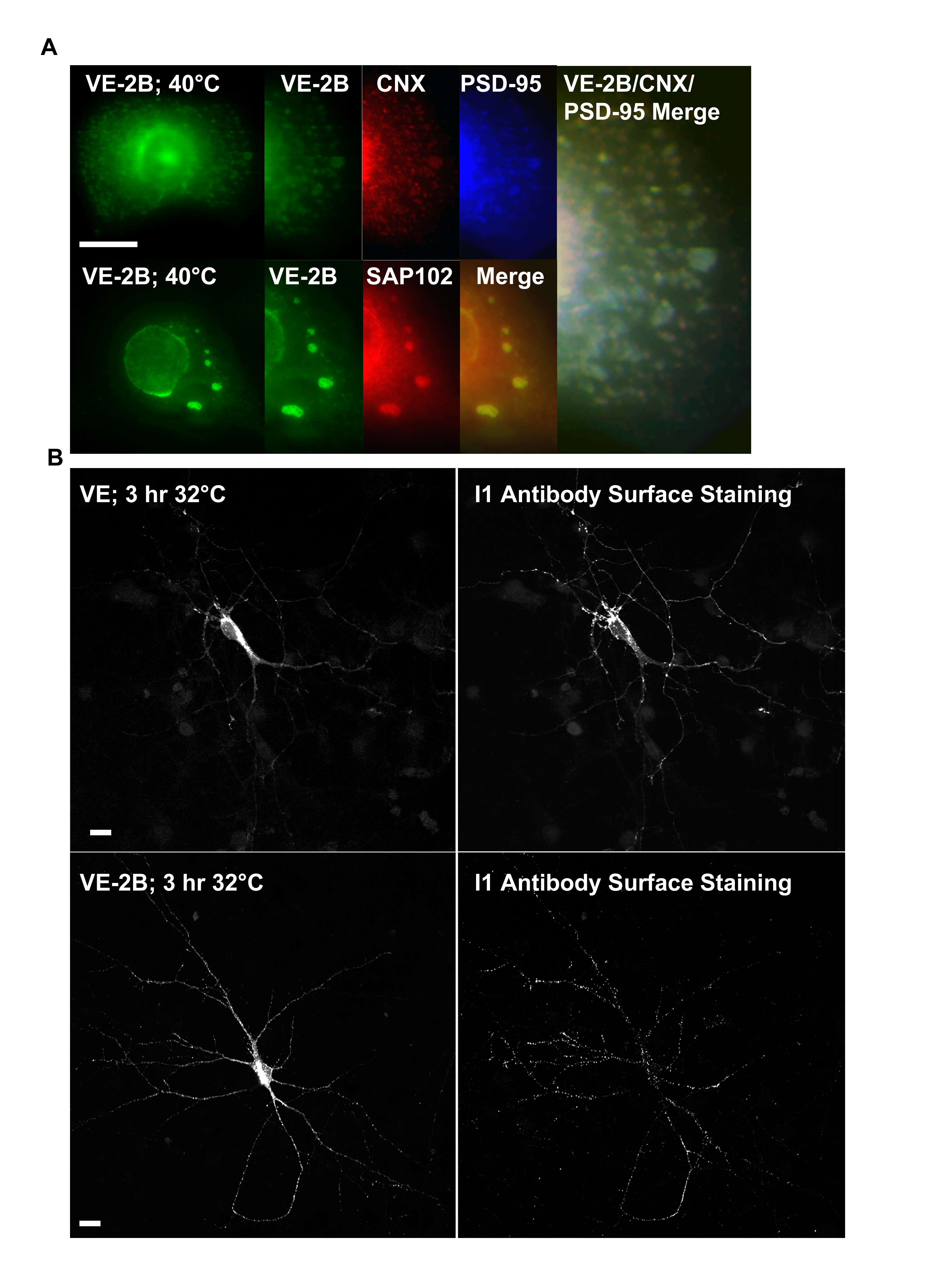

Supplement: Figure S2 — Characterization of VE-NR2 chimeras. (A) VE-2B and PSD-95 co-clustered in the ER in heterologous cells. VE-2B (upper left green panel; scale bar 25 µm) and PSD-95 (upper panel pseudocolored blue, fourth from the left most upper panel) were transfected into COS-1 cells and maintained at 40°C overnight, then immunostained with mouse anti-PSD-95, and rabbit anti-calnexin (CNX; upper red panel, third from the left). The large merged panel (far right panel, predominantly white, indicating 3-color colocalization) clearly indicated VE-2B co-clustered with both PSD-95 and Calnexin at the level of the ER. Moreover, the clustering appeared similar to prior examples of PSD-95 clustering at the plasma membrane [75]. VE-2B also co-localized with SAP102 when maintained at 40°C (lower four panels from left to right are VE-2B, followed by enlarged VE-2B, SAP102, and Merge). We noted, however, that SAP-102 did not induce clustering per se, but showed the same pattern of co-localization accumulated on the nuclear membrane and in intracellular perinuclear structures as has been previously noted when SAP102 was co-expressed with another receptor binding partner, Kv1.4, which resulted in an intracellular accumulation of both proteins, and an absence of surface targeting [75]. (B) VE is normally added in a constitutive fashion to the cell surface in other cell types (see for example, [76]), and appeared to exhibit the same characteristic in neurons. At 3 hours of permissive temperature, VE alone covered the entire surface of the neuron and exhibited a relatively equal distribution throughout (compare the left upper panel EGFP fluorescence of VE to the I1 surface staining in the upper right panel; scale bars, 10 µm). In comparison, much of VE-2B appeared to remain intracellular (compare the lower left panel to lower right panel), and the neuronal-surface VE-2B was limited to discrete clusters even after 3 hours at permissive temperature. VE and VE-2B images were processed in the same mann [file pone.0039585.s002.tif]

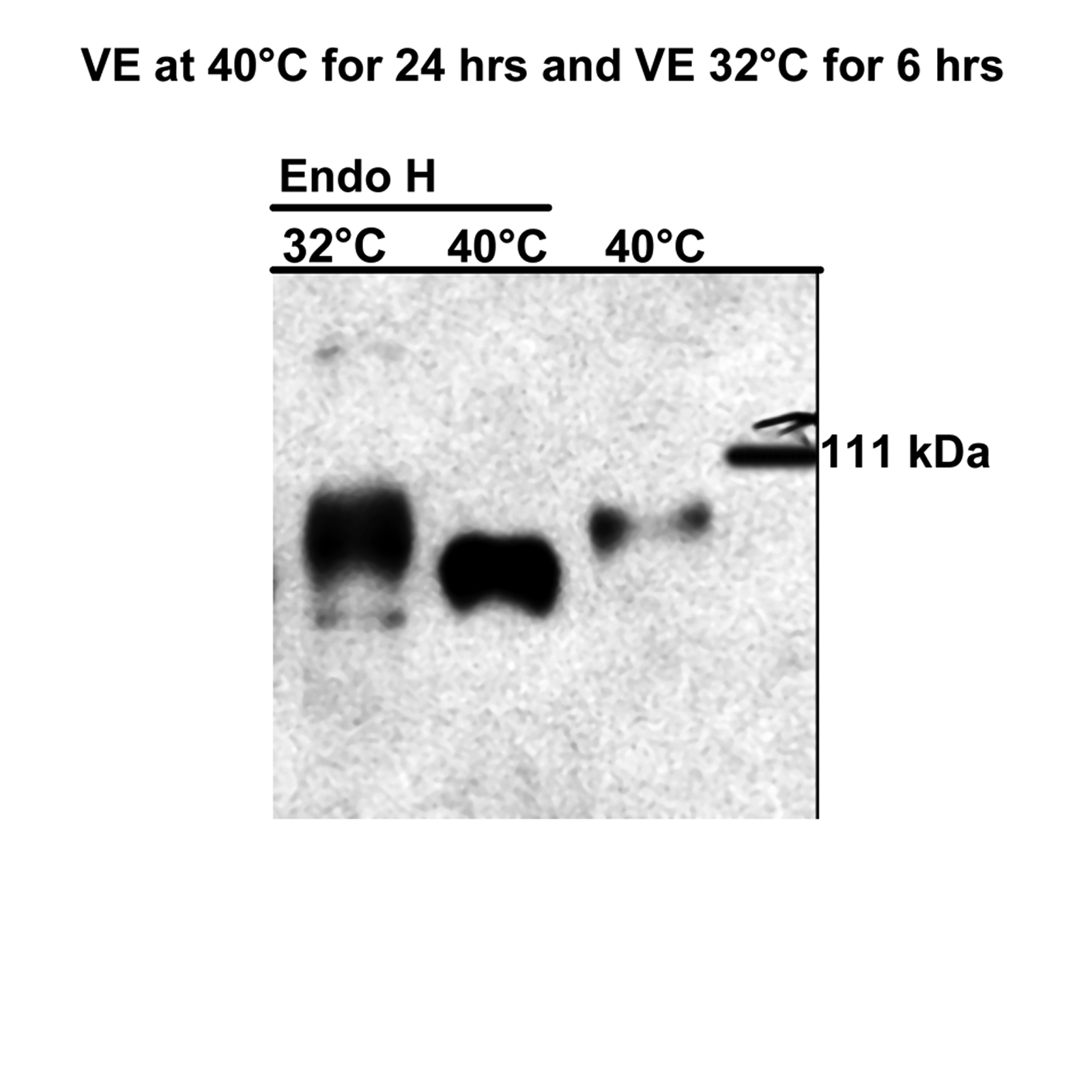

Supplement: Figure S3 — VE is tightly restricted to the ER when maintained at 40°C for 24 hrs. VE was transfected into COS-1 cells for 8 hours, then incubated overnight at 37°C (approximately 16 hours), then placed in a culture incubator equilibrated at 40°C for 24 hrs. Media was preincubated to 32°C, and then the 40°C media was rapidly switched out for the 32°C media. Trafficking of VE was terminated by placing the tissue culture plates on ice after 6 hours at 32°C. Plates were scraped of cells and prepared for Endoglycosidase H (Endo H) treatment and gel electrophoresis. Samples were loaded into a polyacrylamide gel with molecular weight standards, and transferred to membranes using standard methods. Note that the 40°C, 24 hour sample is completely Endo H sensitive (second band from the right). Note also that the small lower molecular weight band in the 32°C lane is a non-specific band that is seen at 32°C but not in samples incubated with or without Endo H at 40°C. Further, N-Glycosidase F treatment, which cleaves all forms of N-glycosyl moieties, migrates no lower than the Endo H-sensitive band (data not shown). (TIF) [file pone.0039585.s003.tif]
