# Supplementary material for: Trafficking of the NMDAR2B Receptor Subunit Distal Cytoplasmic Tail from Endoplasmic Reticulum to the Synapse
Source: PLoS One. 2012 Jun 27;7(6):e39585. doi: 10.1371/journal.pone.0039585 (PMC3384676; doi:10.1371/journal.pone.0039585)
Supplement: Table S1 — Summary of co-localization and targeting of VE constructs. Percent overlaps with PSD-95, SAP102, GM130 and TGN38 are given at 4X background, while overlap of VE constructs with synaptophysin is given at 2X background. Asterisks indicate significance after one-way Anova, with post hoc comparisons using the Tukey method. (DOCX) [file pone.0039585.s004.docx]

| **Construct** | **Time at permissive Temp** | **PSD-95** | **SAP102** | **Synaptophysin** | **GM130** | **TGN38** |
| --- | --- | --- | --- | --- | --- | --- |
| VE | 45 minutes | N/M | N/M | N/M | N/M | N/M |
| VE | 3 hours | N/M | 16.0±3.4 | 25.7±0.06 | N/M | N/M |
| VE-2A | 10 minutes | N/M | 57.4±14.3* | N/M | N/M | N/M |
| VE-2A | 45 minutes | 38.3±7.6* | N/M | N/M | N/M | N/M |
| VE-2A | 3 hours | 31.9±3.8* | 40.7±4.6* | 34.8±2.6 | N/M | N/M |
| VE-2B | 10 minutes | 22.5±1.8 | 56.2±4.2* | N/M | 54.8±7.2 | N/M |
| VE-2B | 45 minutes | 43.7±3.4* | 59.7±3.8* | N/M | N/M | N/M |
| VE-2B | 3 hours | 43.7±10.8* | 45.4±4.8* | 48.7±4.1* | N/M | N/M |
| VE-2B | 1 hour at 20˚C | 39.3±4.8 | N/M | N/M | N/M | 64.0±5.1 |
| VE-2BΔ7 | 10 minutes | 16.7±3.4 | N/M | N/M | N/M | N/M |
| VE-2BΔ7 | 45 minutes | N/M | 11.3±5.2 | N/M | N/M | N/M |
| VE-2BΔ7 | 3 hours | 19.9±5.3 | 19.2±3.7 | 49.2±4.3* | N/M | N/M |

Table S1: Summary of Trafficking and Targeting Properties

*Indicates significant colocalization compared to control
